# Towards Lightweight and Multi-Scale Scene Classification: A Lie Group-Guided Deep Learning Network with Collaborative Attention

**DOI:** 10.3390/jimaging12030094

**Published:** 2026-02-24

**Authors:** Xuefei Xu, Chengjun Xu

**Affiliations:** 1 School of Information Engineering, Shanghai Dianji University, Shanghai 201306, China; xuxf@sdju.edu.cn; 2 School of Artificial Intelligence, Jiangxi Normal University, Nanchang 330022, China; 3 School of Remote Sensing and Information Engineering, Wuhan University, Wuhan 430072, China

**Keywords:** attention mechanism, feature fusion, Lie Group machine learning, remote sensing scene classification

## Abstract

Remote sensing scene classification (RSSC) plays a crucial role in Earth observation. Current deep learning methods, while accurate, tend to focus on high-level semantic features and overlook complementary shallow details such as edges and textures. Moreover, conventional CNNs are limited by fixed receptive fields, whereas transformers incur high computational costs. To address these limitations, we propose the Lie Group lightweight multi-scale network (LGLMNet), a lightweight multi-scale network that integrates Lie Group covariance features. It employs a dual-branch architecture combining Lie Group machine learning (LGML) for shallow feature extraction and a deep learning branch for high-level semantics. In the deep branch, we design a parallel depthwise separable convolution block (PDSCB) for multi-scale perception and a spatial-channel collaborative attention mechanism (SCCA) for efficient global–local modeling. A cross-layer feature fusion block (CLFFB) effectively merges the two branches. Compared with state-of-the-art methods, the proposed LGLMNet achieves accuracy improvements of 2.14%, 2.32%, and 1.12% on UCM-21, AID, and NWPU-45 datasets, respectively, while maintaining a lightweight structure with only 2.6 M parameters.

## 1. Introduction

High-resolution remote-sensing images (HRRSI) are fundamental to Earth observation, providing detailed surface information essential for various applications [1]. Advances in sensor technology have significantly enhanced the spectral and spatial richness of HRRSI [2]. With the growing volume and resolution of such imagery, developing efficient and reliable scene classification methods has become a key research focus [3,4,5].

Remote sensing scene classification (RSSC) is a fundamental task in HRRSI interpretation, aiming to classify HRRSI scenes based on their semantic content [1]. Recent decades have seen the advancement of remote sensing technology along with increased multidisciplinary collaboration, leading to the broad application of RSSC in various fields, including urban planning [6,7], natural disaster detection [8,9], geospatial object detection [10,11], and environmental monitoring [12,13]. RSSC models are especially valuable for urban land-use classification, as the resulting land-use maps provide critical decision support for the effective planning and management of urban areas [14,15].

**Figure 1:**
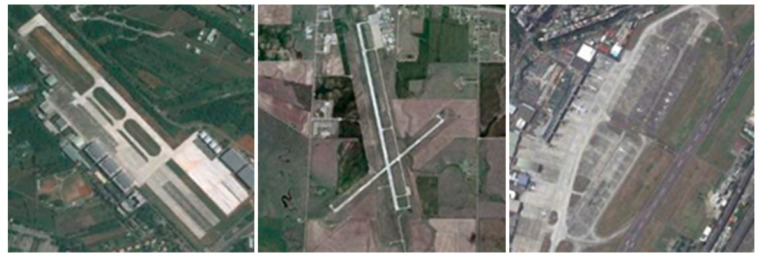
Large variations in scene scale: Example of an airport scene at different scales (images sourced from the NWPU-45 dataset).

**Figure 2:**
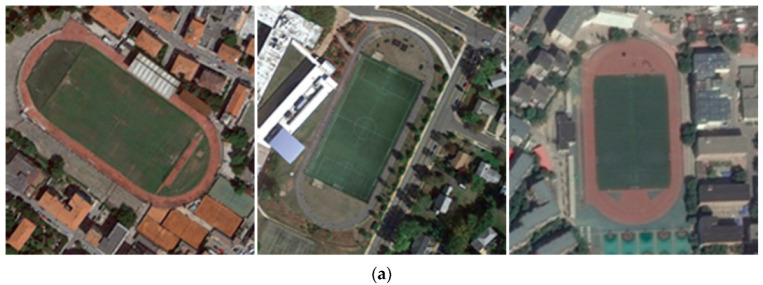

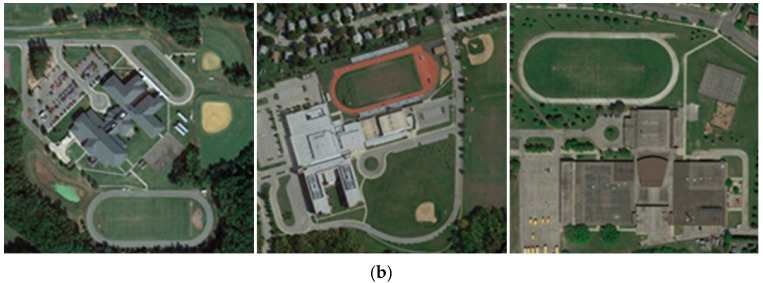
The field of view of HRRSI is typically large, encompassing multiple types of ground objects. Subfigure (**a**) depicts a playground scene, while subfigure (**b**) shows a school scene. (Images are sourced from the AID dataset).

**Figure 3:**
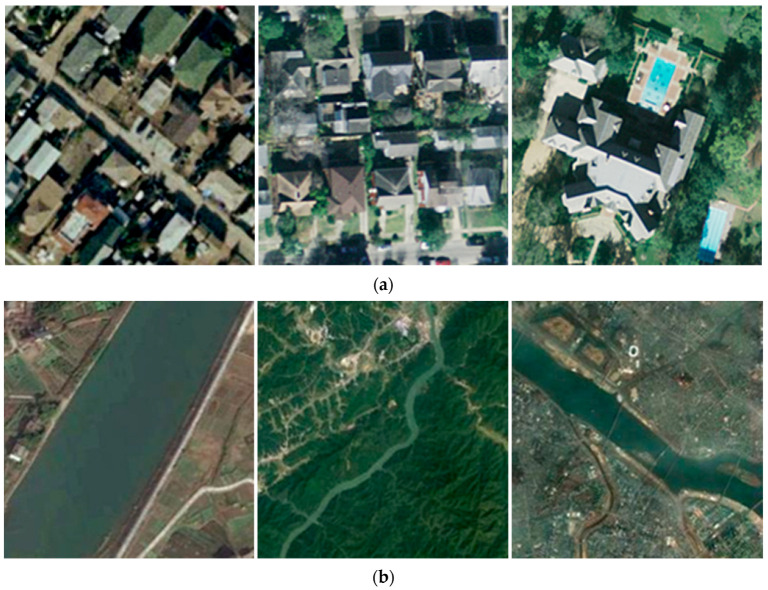
Resemblance among classes and notable diversity within classes: (**a**) The three scenes, from left to right, depict dense, medium, and sparse residential. (**b**) From left to right are images of the same scene category: river (Images are sourced from the UCM and NWPU-45 datasets).

In comparison with natural image classification, RSSC presents a more substantial set of challenges attributable to the following characteristics of HRRSI:

1. Large variation in scene scale: Remote sensing sensors are deployed on platforms whose altitudes range from a few hundred to tens of thousands of kilometers; their imaging modes, spatial resolutions, and viewing geometries likewise vary, and these factors jointly determine image scale [1]. Take an airport scene as an example (as shown in Figure 1); the same scene target presents different scale features under different imaging heights and sensor parameters. In addition, the inherent spatial size differences in various targets in the scene further expand the scale distribution in the dataset.

2. The field of view of HRRSI is typically large, encompassing multiple types of ground objects. Due to the wide coverage of HRRSI and the complex spatial distribution of ground objects, a scene often contains multiple types of land cover features. This complexity increases the difficulty of extracting key discriminative features from HRRSI, thus making RSSC more challenging. As shown in Figure 2, the playground scene in (a) contains elements such as athletic fields, buildings, and trees, while the school scene in (b) contains similar elements such as athletic fields, buildings, and trees. In this case, identifying and extracting the most discriminative features becomes particularly difficult.

3. Resemblance among classes and notable diversity within classes: In different HRRSI scenes, similar or identical ground objects often appear in different classes, leading to indistinguishable high-level semantic features and increasing the difficulty of accurate categorization. For example, as shown in Figure 3a, the dense residential scene, the medium residential scene, and the sparse residential scene all contain similar objects, such as houses, trees, and roads. Figure 3b illustrates three river scenes at varying scales, clearly demonstrating the high intra-class variation within the same HRRSI category, which is attributed to factors such as object distribution, scale, and sensor differences.

In recent years, deep learning (DL) methods have experienced rapid advancements, prompting researchers to apply them to address challenges in RSSC [16,17,18]. Convolutional neural networks (CNNs) are widely adopted in RSSC due to their ability to extract discriminative features from HRRSI automatically. Consequently, scholars have proposed numerous CNN-based RSSC models [19,20,21]. For instance, Xu et al. [21] developed the Lie Group regional influence network (LGRIN) by merging Lie Group machine learning with CNN. While CNNs excel at extracting local features but struggle with long-range dependencies, researchers proposed the vision transformer (ViT) model, which learns the contextual features of images and captures the relationships between different spatial regions [22,23,24]. However, ViTs address global context at the cost of high computational complexity and often underperform on fine local details [25,26,27,28].

Although the above models have achieved some progress in RSSC, several challenges remain:

1. Missing shallow features: Shallow features refer to low-level and mid-level features that capture fine spatial details before deep semantic abstraction, including edges, gradients, colours, and local textures (such as the scale-invariant feature transform (SIFT) [29] and local binary pattern (LBP) [30]), as well as Gabor responses, etc. CNN and transformer-based RSSC models primarily rely on convolution to extract advanced semantic information from the scene, often neglecting shallow features (low-level and mid-level) [31]. While shallow features are important for detail capture in HRRSI, for example, there are significant differences in edge texture and local patterns between the two types of scenes, namely, residential areas and churches, which play a crucial role in scene representation and classification performance [32].
2. Difficulties in combining local and global features: CNNs are effective at extracting local features but face challenges in capturing large-scale spatial relationships and long-range dependencies in HRRSIs [33]. Although ViT is capable of capturing global contextual information, it underperforms on small-scale local features and is especially prone to ignoring critical local details in HRRSI processing [34]. Therefore, our model should fully consider the fusion of local and global features.
3. High computational complexity and multiple model parameters: To achieve high classification accuracy, existing CNNs typically employ stacked convolutional operations, which can increase model complexity and require multiple model parameters [35]. Furthermore, the transformer model’s high computational complexity restricts its practical application to a limited range of scenarios, making deployment on devices with limited resources challenging [36].

Unlike recent state-space models (e.g., RSMamba [37]) that focus on efficient global modeling, or existing Lie Group machine learning (LGML)-CNN hybrids [21,36] that may not fully optimize multi-scale local extraction and cross-layer fusion, our paper proposes a lightweight multi-scale convolutional neural network that incorporates Lie Group features: Lie Group lightweight multi-scale network (LGLMNet). LGLMNet simultaneously and explicitly addresses the above three challenges.

The contributions of this work are as follows:

1. To address the first challenge above, the model effectively preserves the shallow features (including low-level and mid-level features) of HRRSI, such as the SIFT [29] and LBP [30], through LGML. Meanwhile, the model utilizes DL to extract high-level semantic features from HRRSI, thereby comprehensively capturing the complex information in the scene.
2. To address the second challenge above, based on previous research, we propose the cross-layer feature fusion block (CLFFB), which fully exploits the correlation between shallow and high-level features, thereby reducing redundancy. Furthermore, we integrate the strengths of CNN and transformer to develop the spatial-channel cooperative attention mechanism (SCCA), enhancing both local and global semantic connections. Meanwhile, key information from early layers is retained through residual connections, strengthening the model’s robustness.
3. To address the third challenge above, our model adopts a lightweight design. Specifically, in the DL branch, inspired by InceptionNet [35], we design the parallel depthwise separable convolution block (PDSCB). In addition, the SCCA attention mechanism proposed in this paper employs operations such as multi-receptive field shared depthwise convolutions and linear self-attention. The methods mentioned above effectively reduce the dimensionality of features and the number of model parameters, thereby enhancing feature expressiveness and lowering computational complexity.

## 2. Related Work

### 2.1. RSSC Methods

RSSC methods can be categorized based on the level of extracted features: low-level, mid-level, and high-level [38]. These categories are not mutually exclusive and often overlap in practice. To clearly position our work, we review RSSC methods through the lens of feature abstraction levels, as the evolution from low-level to high-level features reveals both the progress made and the persistent challenges. Specifically, we discuss how methods at each level have inherent limitations that collectively motivate the integrated and lightweight design of our proposed LGLMNet.

#### 2.1.1. Methods Dominated by Low-Level Features

Methods dominated by low-level features were the early foundational methods for RSSC, relying on low-level visual features of images, such as texture, shape, color, and edges [39]. By directly analyzing pixel-level information, these methods extract features using mathematical descriptors such as SIFT [29], LBP [30], and the grey level covariance matrix (GLCM) [40] and classify them utilizing conventional classifiers such as support vector machines (SVM) or the K-nearest neighbor algorithm (KNN). For example, Huang et al. [41] proposed a method based on multi-scale complete local binary pattern (MS-CLBP) features of patches and Fisher vector (FV). Risojević et al. [42] utilized SVM for classifying land use in HRRSI using Gabor and Gist descriptors. Kabir et al. [43] introduced a technique that utilizes GLCM and the maximum likelihood method to collect data on urban land cover and land use. Such methods are more effective in situations with consistent structures and spatial rules. Nevertheless, it has a limited effect in situations involving complex geometric shapes and increased spatial variation, as it relies on experts’ prior knowledge, which makes it difficult to manage complex scenarios [44].

These methods demonstrate the importance of shallow, hand-crafted features for basic scene discrimination. However, their reliance on fixed descriptors and prior knowledge limits their ability to handle complex semantics and large variations in modern HRRSI. This highlights the value of shallow features but also underscores the need for a more adaptive and integrated approach to leverage them, which motivates our dedicated LGML branch.

#### 2.1.2. Methods Dominated by Mid-Level Features

To overcome the shortcomings of low-level feature methods, researchers have proposed methods dominated by mid-level features. By further processing low-level features and extracting higher-order statistical information, mid-level features can capture key patterns and construct more complete representations, and commonly used methods include bag of visual words (BoVW) [45], probabilistic topic models (PTM) [46], and Fisher’s kernel (FK) [47], etc. For example, Zhu et al. [48] proposed a local-global-featured BoVW scene classifier (LGFBoVW) by introducing a global shape histogram based on the traditional SIFT-BoVW approach. Zhao et al. [47] proposed a local Fisher kernel (LFK) for RSSC based on the distributional differences in ground targets in various HRRSI regions. Zhong et al. [49] proposed a PTM-based semantic assignment level (SAL) multi-feature fusion strategy (SAL-PTM) for RSSC. Although mid-level features have made progress in enhancing expressiveness, their limitations lie in ignoring inter-feature correlation [50], relying on domain knowledge and parameter tuning [51]. Furthermore, in HRRSI with complex geometries and high spatial variability, performance is limited, and it is difficult to fully capture global semantics and deep associations.

Mid-level feature methods offer a more compact representation but often rely on complex encoding processes and still struggle with capturing deep semantic relationships and global context. This gap between manually engineered mid-level representations and data-driven high-level semantics informs our design of a CLFFB to bridge shallow and deep features in a learnable manner.

#### 2.1.3. Methods Dominated by High-Level Features

Methods that primarily utilize high-level features, often employing DL techniques such as CNN and transformer, autonomously derive high-level semantic attributes from HRRSI, thus overcoming the limitations of traditional feature design [39]. For example, Song et al. [52] proposed a network for multi-scale feature fusion of CNN and transformer (CTMFNet) for remote sensing interpretation of urban scenes. Zhao et al. [53] proposed a progressive aggregation method for RSSC based on the synergistic learning of local and global features. Yu et al. [54] designed the spectral-spatial transformer for hyperspectral image classification. Chen et al. [55] proposed a deep nearest neighbor neural network (DN4AM) based on an attention mechanism for the small-sample RSSC. Zhao et al. [56] designed an enhanced attention module (EAM) for RSSC to improve the feature extraction and generalization ability of deep neural networks. Chen et al. [37] designed RSMamba based on a state space model (SSM), which showed outstanding results across multiple HRRSI datasets. These methods perform well in feature extraction but are prone to overfitting when there is insufficient data or sample imbalance, and they rely on large-scale, labelled data and substantial computational resources [1].

While DL methods have become dominant, their focus on high-level semantics often comes at the expense of discarding low-level details, and their model complexity can be prohibitive. Moreover, the trade-off between CNN’s local focus and Transformer’s global modeling remains a key issue.

### 2.2. LGML

LGML is a developing area in machine learning, fundamentally rooted in the Lie Group mathematical framework. This framework forms an innovative learning paradigm by leveraging the geometric advantages of the manifold structure [57]. Scholars have introduced LGML into RSSC. For example, Xu et al. [58] propose an algorithm that utilizes the mean within the Lie Group and develops the Lie Group kernel function. This algorithm effectively processes both matrix and vector data samples, showcasing its versatility, and has achieved strong classification results in HRRSI datasets. Subsequently, Xu et al. [59] refined the algorithm by optimizing the model’s structure and reducing the parameter count, resulting in a significant improvement in classification accuracy. In addition, Xu et al. [21] introduced an RSSC model that integrates Lie Group features with CNN, enhancing classification accuracy and interpretability while retaining key image information more effectively. To successfully blend global and local multi-scale features, Xu et al. [36] proposed a model that integrates global and local features with attention in Lie Group space.

LGML excels in robustness to orientation changes and geometric transformations. Lin et al. [60] verified its adaptability to orientation changes and extended the application scenarios of geometric modelling by introducing Lie groups and Lie algebras in affine transformations. Huang et al. [61] modelled the motion patterns of key points of the skeleton for action recognition, behavioral analysis, and dynamic gesture recognition, demonstrating. Xu et al. [62] proposed a Lie-X framework based on Lie groups for pose estimation, target tracking, and action recognition in in-depth images, which unifies the modeling of rigid and non-rigid motions.

The core rationale for adopting LGML in our framework lies beyond its mere application in prior works. The key advantage is its mathematical foundation for representing an ensemble of heterogeneous features. By constructing a symmetric positive definite (SPD) covariance matrix from low/mid-level feature vectors (e.g., SIFT, LBP, gradients), LGML embeds the data onto a Riemannian manifold. This representation offers two critical benefits over flat vector concatenation or traditional statistical pooling: (1) It intrinsically encodes the second-order correlations between different feature types, capturing richer structural information about the scene. (2) The geometry of the SPD manifold provides a natural framework for achieving robustness to certain geometric variations, which is highly desirable in RSSC [57,59]. Therefore, we employ LGML not only as a feature extractor but as a principled shallow feature integrator and robustifier within our dual-branch architecture.

## 3. Methods

### 3.1. Overview

**Figure 4:**
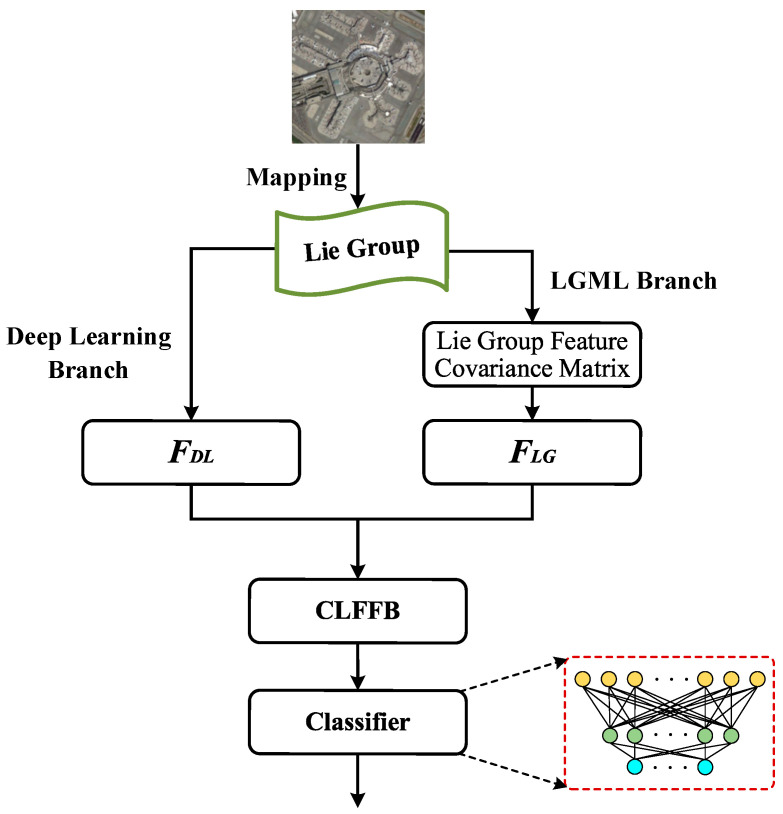
Network structure of the method proposed in this paper (LGLMNet), including (1) LGML branch: for extracting shallow features, F_LG_ represents the shallow Lie Group features extracted by LGML; (2) deep learning Branch: for extracting high-level semantic features, F_DL_ represents the high-level semantic feature extracted from the deep learning branch; (3) CLFFB: for features fusion; and (4) classifier: composed of a MLP, is used to map the feature tensor to a dimension equal to the number of categories.

A novel model, termed the LGLMNet, is proposed in this study. It is a lightweight, multi-scale convolutional neural network that integrates features from Lie Group, as illustrated in Figure 4. The model’s architecture includes three core components: the LGML branch, the DL branch, and the CLFFB. The LGML branch involves extracting the shallow features of HRRSI and utilizing the Lie Group feature covariance matrix for feature representation. In the DL branch, we designed a PDSCB and an SCCA to extract high-level features. Finally, CLFFB integrates the features extracted from the LGML and DL branches and employs a multi-layer perceptron-based (MLP) classifier to perform the final classification. The classifier is designed based on a multi-layer perceptron, which is mainly based on the following considerations: MLP can flexibly map the fused high-dimensional features to the category space. Its structure is simple, and the number of parameters is small, which is in line with the overall design principle of lightweighting the model. At the same time, the feature vectors after global average pooling are suitable as the input of the MLP. This combination has been widely verified in remote sensing scene classification.

### 3.2. LGML Branch

This branch primarily focuses on extracting shallow scene features, with the learning of discriminative features being a key aspect of RSSC. In previous studies, scholars have found that in some scenes lacking significant target objects, such as deserts, fields, and forests, selecting shallow and mid-level features tends to achieve better classification results than selecting high-level semantic features [38]. To improve the model’s classification performance in the previously mentioned scenarios, we extracted both low-level and medium-level features from the scenes in the LGML branch. We generated feature maps using the Lie Group covariance matrix with symmetry.

#### 3.2.1. Mapping of Sample Data

In order to better exploit the advantages of Lie groups and Lie algebras in terms of computation and the structure of manifold spaces, it is first necessary to map the data samples into Lie Group manifold spaces:

(1) $$G_{ij}=\log\left(S_{ij}\right)$$

where $S_{ij}$ indicates the j-th sample of the i-th class in the dataset, while $G_{ij}$ represents the corresponding sample in the Lie Group manifold space. All following operations are executed using the samples $G_{ij}$ in the Lie group manifold space [59]. Here, $S_{ij}\in R^{d\times d}$ is the covariance matrix computed from the feature vector F(x,y) (Equation (2)) for the given sample. Covariance matrices are Symmetric Positive Semi-Definite (SPSD). To ensure they are strictly SPD for a valid log-map operation on the manifold, we add a small regularization term ε (where ε = 1 × 10^−6^) during computation, a common practice in Riemannian geometry [59].

#### 3.2.2. Lie Group Covariance Matrix

To capture more discriminative features, we extracted a range of low-level and mid-level features as follows:

(2) $$\begin{array}{c} F\left(x,y\right)=[x,y,\left|\frac{\partial I\left(x,y\right)}{\partial x}\right|,\left|\frac{\partial I\left(x,y\right)}{\partial y}\right|,\left|\frac{\partial^{2}I\left(x,y\right)}{\partial x^{2}}\right|,\left|\frac{\partial^{2}I\left(x,y\right)}{\partial y^{2}}\right|,\\ {SIFT\left(x,y\right),LBP\left(x,y\right),Gabor\left(x,y\right),Y,C_{b},C_{r}]}^{T} \end{array}$$

where (x,y) denotes the position of the target image element in the scene, and $\left(\left|\frac{\partial I\left(x,y\right)}{\partial x}\right|,\left|\frac{\partial I\left(x,y\right)}{\partial y}\right|),(\left|\frac{\partial^{2}I\left(x,y\right)}{\partial x^{2}}\right|,\left|\frac{\partial^{2}I\left(x,y\right)}{\partial y^{2}}\right|\right)$ denote the first and second derivatives of the coordinate position  (x,y), respectively, which are capable of more finely capturing the local variation and boundary information. In order to extract the texture information of local regions, we employ $SIFT\left(x,y\right)$ and $LBP\left(x,y\right)$ features. $SIFT\left(x,y\right)$ [29] can capture the texture information of local key points with good scale, rotation and illumination invariance, which makes it able to cope with the viewpoint variations that are commonly found in remote sensing images. $LBP\left(x,y\right)$ [30], on the other hand, describes the details of local regions by binarizing the texture, is robust to illumination variations, and can effectively distinguish between different types of feature textures. $Gabor\left(x,y\right)$ is a linear filter that can mimic the receptive fields of cortical neurons. It effectively captures local features, including textures and edges, within an image. [63,64]. The luminance component Y, and chrominance component $C_{b},C_{r}$ of the $Y,C_{b},C_{r}$ colour space are also introduced to enhance the colour discrimination ability in the classification task. Compared with the RGB space, the $Y,C_{b},C_{r}$ space can separate the luminance and colour information more efficiently, and exhibits a stronger discriminative ability in distinguishing between different feature types (e.g., forest, beach, desert) [65]. This multi-dimensional feature combination fully contains shallow features (e.g., edges, gradients, colors) and intermediate features (e.g., texture, local structure), comprehensively characterizes remote sensing scene properties from multiple levels, effectively improves the robustness and accuracy of scene classification, and provides strong support for complex scene classification [1].

This multi-dimensional feature combination is designed to comprehensively capture scene properties: spatial coordinates and derivatives provide layout and edge information. SIFT and LBP offer complementary texture descriptions robust to scale and illumination, Gabor filters capture oriented frequency patterns, $Y,C_{b},C_{r}$ color space separates luminance and chrominance for better color discrimination. While high-dimensional, these features are not used directly. Instead, their intercorrelations are compactly encoded into a covariance matrix, which serves as a robust and low-dimensional descriptor on the Lie Group manifold.

### 3.3. DL Branch

**Figure 5:**
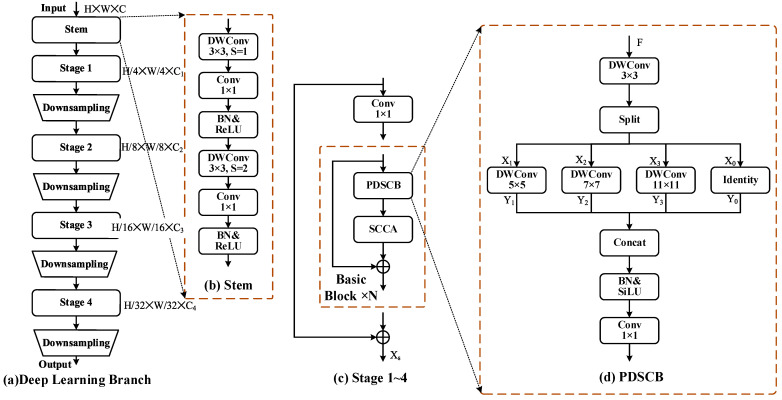
(**a**). The general structure of the DL branch, including Stem, Stage, and Downsampling components. (**b**). The structure of Stem, which mainly consists of two layers of depthwise separable convolutions, where S represents the stride. (**c**). The structure of Stages 1~4, SCCA denotes the spatial-channel collaborative attention mechanism, ⨁ denotes element-wise addition of the tensors from two branches (**d**). The structure of the PDSCB, Split denotes the partitioning along the channel dimensions, DWConv denotes the depthwise separable convolution, and Concat denotes the concatenate operation, which merges multiple feature tensors by concatenating them along a designated dimension. BN denotes batch normalization, and SiLU is the activation function.

The DL branch primarily captures high-level features of images, which contain richer semantic information and effectively mitigate the influence of background noise and other interfering factors. The structure of the DL branch is illustrated in Figure 5a, where the input image passes through the Stem layer consisting of two layers of 3×3 depthwise separable convolutions (As shown in Figure 5b), generating the base feature map. Subsequently, it passes through Stages 1 to 4 sequentially, and each Stage contains several Basic Blocks (As shown in Figure 5c). Downsampling is performed by maxpooling between neighboring Stages, gradually expanding the receptive field and compressing the computation, and finally outputting feature maps with rich high-level semantic information after Stage 4.

#### 3.3.1. PDSCB

To capture more scene contextual feature information in remote sensing images, existing methods often employ the strategy of large kernel convolution or dilation convolution to expand the receptive field, accommodating a wide range of scene scales and diverse target features. However, large kernel convolutions often introduce considerable noise and computational complexity, potentially missing the learning of small-scale features and complicating the computational process. The use of dilation convolution increases feature sparsity and also increases computational complexity [66]. Therefore, to efficiently capture multi-scale semantic information of remote sensing images and facilitate feature interaction, this paper proposes a PDSCB. In contrast to techniques that depend on large kernel convolutions or dilated convolutions to increase the receptive field, the convolution layer of PDSCB utilizes a multi-parallel branch depthwise separable convolution kernel, without dilation, to capture dense texture features from image scenes across various receptive fields.

This is shown explicitly in Figure 5d. The module features a four-branch parallel architecture, including a constant mapping branch, and 5×5, 7×7, and 11×11 depthwise separable convolutional branches. Different convolutional branches adopt different sensing fields, which enables the PDSCB module to simultaneously extract multi-scale semantic information of small targets (buildings, aircraft), mesoscale blocky areas (farmland, forest), and large-scale structures (river, road) in HRRSI. By leveraging the strategies of depthwise separable convolution and multi-scale branches, PDSCB effectively reduces the number of parameters and computational requirements, while enhancing the network’s generalization ability in complex and variable scenarios. Table 1 lists the hyperparameter settings in Figure 5.

Let the input features of the PDSCB be $F\in R^{B\times C\times H\times W}$, where B represents the batch size, C represents the number of channels, H represents the height of the feature, and W represents the width of the feature. F is first extracted from the local features by a 3×3 depthwise separable convolution to obtain X, which is then partitioned along the channel dimensions into four sub-tensor features:


(3) $$X_{0},X_{1},X_{2},X_{3}\;\left(X_{i}\in R^{B\times \frac{C}{4}\times H\times W}\right)=split\left({DWConv}_{3\times 3}\left(F\right)\right)$$

The split(·) function divides the input feature map into four groups along the channel dimension. Subsequently, through four parallel branches:

(4) $$Y_{0}={Identity(X}_{0})$$

(5) $$Y_{1}={DWConv}_{5\times 5}\left(X_{1}\right)$$

(6) $$Y_{2}={DWConv}_{7\times 7}\left(X_{2}\right)$$

(7) $$Y_{3}={DWConv}_{11\times 11}\left(X_{3}\right)$$

where ${DWConv}_{k\times k}$ denotes depthwise separable convolution with k convolution kernel size.

The kernel sizes {5 × 5, 7 × 7, 11 × 11} are selected to explicitly capture small, medium, and large-scale structures commonly found in HRRSI (e.g., vehicles, individual buildings, and farmlands) without resorting to excessively large kernels that are computationally prohibitive. This design is contrasted with two prevalent multi-scale approaches:

Dilated Convolutions and ASPP: While effective in expanding the receptive field with fewer parameters, dilated convolutions sample input features sparsely, which can lead to gridding artifacts and loss of fine-grained local continuity, a critical drawback for texture-rich RS images. ASPP mitigates this by using multiple dilation rates but inherits the fundamental sparsity. Our PDSCB employs standard (non-dilated) depthwise separable convolutions, ensuring dense and contiguous sampling within each receptive field. This preserves detailed local patterns more faithfully, which is paramount for discriminating scenes with similar global layouts but different local textures (e.g., different residential densities).

Inception modules: Traditional Inception modules use parallel standard convolutions of varying sizes, achieving multi-scale perception at high parameter cost. PDSCB innovates by marrying the multi-branch philosophy with extreme parameter efficiency via depthwise separation. For a feature map with C channels, a K×K depthwise separable branch in PDSCB has only $K^{2}\times C/4$ parameters per spatial location (after channel splitting), compared to $K^{2}\times (C/4)\times C$ for a standard convolutional branch in Inception. This enables PDSCB to offer rich multi-scale representation with minimal parameter growth.

In the channel dimension, we concatenate the features $Y_{0},Y_{1},Y_{2},Y_{3}$ obtained from the four branches, and then after the BN and SiLU activation functions, we further extract the links between the channel features by a 1×1 convolution to obtain the output feature $Z_{0}$ of the PDSCB.

(8) $$Z_{0}={Conv}_{1\times 1}\left(SiLU\left(BN\left(Concat\left[Y_{0},Y_{1},Y_{2},Y_{3}\right]\right)\right)\right)$$

The input feature F is connected to $Z_{0}$ through a residual connection, and then the final output feature Z of the Basic Block is obtained through the SCCA attention mechanism.

(9) $$Z=SCCA\left(Z_{0}\right)+F$$

PDSCB constructs scene-rich and robust multi-scale semantic features while reducing the feature dimensions. As shown in Figure 5c, within each Stage, firstly, the feature $X{\in R}^{B\times C\times H\times W}$ is expanded the number of channels by 1×1 convolution. Then the output feature is obtained through N Basic Blocks, which are residually connected with X to prevent the loss of the original important features, and finally, the output $X_{s}$ of the Stage are obtained.

(10) $$X_{s}={BasicBlock}_{\times N}\left(X\right)+X$$

The residual connections are employed throughout the DL branch to mitigate gradient vanishing/explosion issues and ease the learning of identity mappings, which empirically enhances training stability and convergence.

#### 3.3.2. SCCA

**Figure 6:**
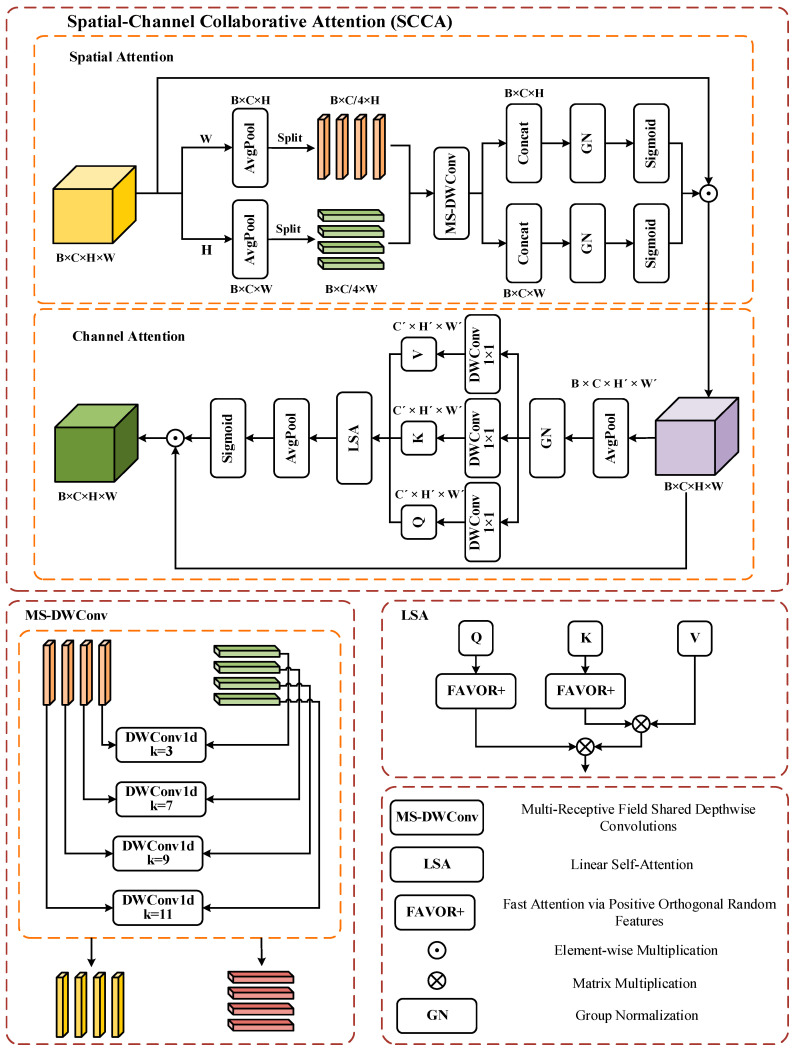
The structure of the SCCA mechanism consists of two parts: spatial attention and channel attention. Here, MS-DWConv denotes multi-receptive field shared depthwise convolutions, and Concat denotes the concatenation operation, which merges multiple feature tensors by concatenating them along a designated dimension. LSA denotes linear self-attention, GN denotes group normalization, Q/K/V respectively represent Query (query vector), Key (key vector), and Value (value vector), which are three groups of vectors used in the self-attention mechanism to calculate correlations. Sigmoid represents the activation function. ⨀ Denotes element-wise multiplication, ⊗ denotes matrix multiplication, and FAVOR+ denotes fast attention via positive orthogonal random features.

The HRRSI typically contains complex, multi-scale information, with features exhibiting differing spatial distributions. Although traditional CNNs can extract local features, they are challenging to capture global contextual information. To enhance models of both global and local contextual dependencies in images, thereby improving the accuracy of the RSSC, inspired by Si et al. [67], we propose the SCCA. SCCA enhances the feature learning capability of the model by combining the two key modules of spatial attention and channel attention, thereby leveraging their respective strengths. Additionally, it introduces linear self-attention (LSA) to reduce computational complexity and memory consumption, as illustrated in Figure 6.

The goal of spatial attention is to weight the input feature maps across the spatial dimension, enabling the model to concentrate on significant areas of the image while diminishing the impact of unimportant regions. For a given input feature map $X\in R^{B\times C\times H\times W}$. Perform average pooling on X along the height and width dimensions to obtain spatial averages $x_{h}$ and $x_{w}$. The pooled features $x_{h}$ and $x_{w}$ are divided into four parts, and then, four depthwise separable convolutions of different dimensions, $k_{i}\in \{3,7,9,11\}$, are applied to extract the spatial features at different scales, and the GN and Sigmoid are applied to obtain the weights $x_{h}^{attn}$ and $x_{w}^{attn}$:

(11) $$\begin{array}{c} x_{h}^{attn}=Sigmoid(GN(Concat({Conv}_{3\times 3}\left(x_{h}\right),{Conv}_{7\times 7}\left(x_{h}\right)\\ \;\;,{Conv}_{9\times 9}\left(x_{h}\right),{Conv}_{11\times 11}\left(x_{h}\right)))) \end{array}$$

(12) $$\begin{array}{c} x_{w}^{attn}=Sigmoid(GN(Concat({Conv}_{3\times 3}\left(x_{w}\right),{Conv}_{7\times 7}\left(x_{w}\right)\\ \;\;,{Conv}_{9\times 9}\left(x_{w}\right),{Conv}_{11\times 11}\left(x_{w}\right)))) \end{array}$$

These features are then fed into the attention mechanism described in Equations (13)–(16). Ultimately, the feature map $x_{sp}$, weighted by spatial attention, is derived from $x_{h}^{attn}$ and $x_{w}^{attn}$, where ⊙ denotes element-wise multiplication:

(13) $$x_{sp}=x_{h}^{attn}⊙x_{w}^{attn}⊙X$$

The second stage of SCCA performs channel attention, which aims to weight each channel so that the model can focus on key features of the channels. Average pooled downsampling of the input feature $x_{sp}$ with a window number of s yields the output $y\in R^{B\times C\times H^{’}\times W^{’}}$, where $H^{’}=H/s$ is the height of the downsampled and $W^{’}=W/s$ is the width of the downsampled feature.

Next, feature y is convolved to generate queries (Q), keys (K), and values (V), and the channels are redistributed to dimension (B,h,N,d) by multi-head attention, where h is the number of heads of the multi-head attention, and $N=H^{’}W^{’}$ is the length of the sequence after downsampling, so that each head of the attention focuses on a subspace of d=C/h features:

(14) $$\begin{array}{c} Q={reshape}_{h}\left({DWConv}_{1\times 1}^{Q}\left(y\right)\right),\\ K={reshape}_{h}\left({DWConv}_{1\times 1}^{K}\left(y\right)\right),\\ V={reshape}_{h}\left({DWConv}_{1\times 1}^{V}\left(y\right)\right) \end{array}$$

In SCCA, to improve computational efficiency, we introduce an LSA mechanism in the channel attention stage, thereby reducing computational complexity. Conventional scaled dot product attention requires explicit computation of $softmax\left(\frac{QK^{T}}{\sqrt{d}}\right)V$, which has a correlation matrix size of N×N and a computational complexity of $O(N^{2}d)$. LSA employs a stochastic feature mapping, FAVOR+ [68], which approximates the softmax kernel $e^{QK^{T}}$ as the inner product of two linear mappings, reducing the complexity to O(Nmd) (m is the mapping dimension, approximately linear when m≪N):

(15) $$e^{QK^{T}}\approx \phi \left(Q\right){\phi \left(K\right)}^{T},\phi :R^{d}⟶R^{m},\phi \left(z\right)=\frac{1}{\sqrt{m}}e^{-∥z∥/2}e^{\Omega_{z}}$$

where $\Omega \in R^{m\times d}$ is an orthogonal Gaussian random matrix, and $Q,K\in R^{N\times d}$ denote the Query and Key matrices. ϕ(z) is a mapping function containing a combination of a Gaussian kernel and a linear projection used to approximate the softmax computation. $R^{d}⟶R^{m}$ denotes that ϕ(⋅) maps the dimension from d to m, and the attention can be linearised as:

(16) $$F_{attn}=\frac{\phi \left(Q\right)\left[{\phi \left(K\right)}^{T}V\right]}{\phi \left(Q\right)\left[{\phi \left(K\right)}^{T}1_{N}\right]}$$

where $1_{N}\in R^{N}$ denotes an all-ones column vector used for attention weight normalization. This formulation achieves linearized attention weighting on the value V by first computing ${\phi \left(K\right)}^{T}V$ (aggregating V based on K) and then multiplying it by $\phi \left(Q\right)$. LSA approximates the softmax kernel via random feature mapping ϕ(⋅), reducing computational complexity from $O(N^{2}d)$ to O(Nmd)(m≪N). The approximation provided by methods like FAVOR+ has well-established theoretical error bounds [69]. The adoption of LSA in SCCA is primarily motivated by the need to significantly reduce computational and memory overhead while maintaining the ability to model global context, representing a trade-off between efficiency and performance. We empirically selected the random feature dimension m=64 (with default heads = 4), which was determined as a balance point after comparing m∈{32,64,128}. The final channel attention weight $x_{ch}$ is then obtained by global average pooling (GAP) and the Sigmoid activation function:

(17) $$x_{ch}=Sigmoid(GAP(F_{attn}))$$

The output features of SCCA are finally obtained by combining $x_{sp}$ and $x_{ch}$:

(18) $$X_{out}=x_{sp}⊙x_{ch}$$

By effectively combining spatial and channel attention, SCCA can efficiently capture local details while modelling an extensive range of global contextual information, thus adapting to the wide range of cross-scale and long-distance dependent features in remote sensing images. SCCA adopts linearized attention approximation with default heads = 4 and random feature dimension m=64. We compare m∈{32,64,128} to balance accuracy and latency, and select m=64.

#### 3.3.3. CLFFB

**Figure 7:**
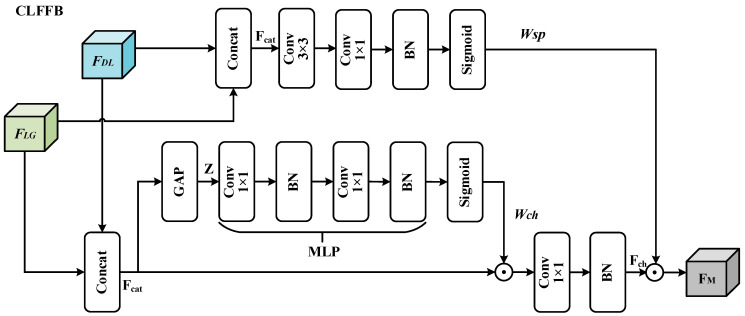
Structure of CLFFB.$F_{LG}$ and $F_{DL}$ represent the output features of the LGML branch and DL branch, respectively. GAP stands for global average pooling, BN stands for batch normalization, Sigmoid denotes the activation function, and Concat denotes the concatenate operation, which merges multiple feature tensors by concatenating them along a designated dimension. $F_{M}$ is the fusion feature, ⊙ denotes element-wise multiplication, $W_{sp}$ and $W_{ch}$ denote the spatial attention weight and the channel attention weight, respectively.

To facilitate the integration of shallow features from the LGML branch and high-level features from the DL branch, we introduce a CLFFB, as shown in Figure 7. CLFFB adaptively recalibrates the feature contributions from the shallow details of the LGML branch and the high-level semantics of the DL branch through learnable channel and spatial attention, thereby producing a more discriminative fused representation.

CLFFB takes as input two feature tensors: $F_{LG}\in R^{B\times C_{1}\times H\times W}$ from LGML and $F_{DL}\in R^{B\times C_{2}\times H\times W}$ from the DL branch. Here, $C_{1}$ and $C_{2}$ denote the channel dimensions of the respective outputs. These features are concatenated along the channel axis to form the fused representation $F_{cat}\in R^{B\times (C_{1}+C_{2})\times H\times W}$:

(19) $$F_{cat}=Concat\left(F_{LG},F_{DL}\right)\in R^{B\times \left(C_{1}+C_{2}\right)\times H\times W}$$

In the channel dimension, the module applies GAP on the fused input $F_{cat}$ to summarize channel-wise information, producing a feature map $Z\in R^{B\times (C_{1}+C_{2})\times 1\times 1}$:

(20) $$Z=GAP\left(F_{cat}\right)\in R^{B\times \left(C_{1}+C_{2}\right)\times 1\times 1}$$

A channel-level attention map is then generated by learning the channel attention weights through a small MLP consisting of a 1×1 convolution:

(21) $$W_{ch}=Sigmoid\left(MLP\left(Z\right)\right)\in R^{B\times \left(C_{1}+C_{2}\right)\times 1\times 1}$$

The concatenated feature map $F_{cat}$ is element-wise multiplied by the channel attention map $W_{ch}$, and then passed through a 1×1 convolution to obtain the channel feature map:

(22) $$F_{ch}={{BN(Conv}_{1\times 1}(F}_{cat}⊙W_{ch}))$$

Along with the spatial dimension, we compute spatial attention weights using 3×3 and 1×1 convolutions. Specifically, we first apply a 3×3 convolution to obtain the spatial features.

(23) $$F_{sp}={Conv}_{3\times 3}\left(F_{cat}\right)\in R^{B\times \left(C_{1}+C_{2}\right)\times H\times W}$$

A 1×1 convolution is subsequently applied to project the spatial features into a single-channel spatial attention map.

(24) $$W_{sp}=Sigmoid\left(BN\left({Conv}_{1\times 1}\left(F_{sp}\right)\right)\right)\in R^{B\times 1\times H\times W}$$

Finally, the spatial attention map $W_{sp}$ is element-wise multiplied with the channel-weighted feature map $F_{ch}$, resulting in the final output feature map $F_{M}$.

(25) $$F_{M}=F_{ch}⊙W_{sp}$$

## 4. Experiments and Analysis

### 4.1. Experimental Datasets

To evaluate the performance and robustness of the proposed approach, three extensively utilized and challenging datasets were employed. Table 2 summarizes the key characteristic parameters of these three datasets.

The detailed introductions of the UCM, AID, and NWPU-45 datasets are as follows:

1. UCM [68]: The UC Merced land use dataset (UCM) was publicly released by the University of California, Merced, in 2010. It was manually cropped from the USGS National Map Urban Scene Orthophotography. The dataset consists of 21 distinct scene categories, each containing 100 high-resolution RGB images with a fixed size of 256 × 256 pixels, resulting in a total of 2100 images. The spatial resolution of each image is approximately 0.3 m. These images represent a diverse set of typical urban scenes, including agriculture, airplane, beach, buildings, dense and sparse residential areas, forest, highway, golf course, and tennis court. In this study, 70% (i.e., 70 images per category) were allocated for training purposes.
2. AID [70]: The Aerial Image Dataset (AID) is a large-scale, multi-source benchmark introduced in 2017. It comprises 10,000 orthorectified RGB images collected from Google Earth, organized into 30 scene categories, with each category containing between 200 and 420 samples. All images are uniformly cropped to a resolution of 600 × 600 pixels, and the ground sampling distance ranges from 0.5 m to 8 m, reflecting variations in sensor platforms and geographic conditions. Captured under diverse seasonal and illumination conditions, AID exhibits rich textural and structural variability, covering representative scenes such as an airport, a farmland, a bridge, a beach, and a railway station. In this study, a 50%/50% train-test split is adopted, with half of the samples from each category randomly selected for training. To ensure fair and consistent comparison with prior works on this dataset, we follow the standard benchmark protocol and adopt a 50%/50% train-test split, as recommended in the original AID publication [71].
3. NWPU-45 [71]: The NWPU-RESISC45 dataset (NWPU-45), released in 2017 by Northwestern Polytechnical University, consists of 31,500 orthorectified RGB images with a fixed size of 256 × 256 pixels. These images are evenly distributed across 45 scene categories, with 700 images per class. The ground sampling distance ranges from approximately 0.2 m to 30 m, reflecting variations in sensor types, geographic conditions, and acquisition settings. Example categories include airport, harbor, forest, and sparse residential areas, each exhibiting significant structural and textural variability. In this study, 70% of the images from each category are randomly selected for training.

### 4.2. Experimental Setup

To mitigate overfitting, various data augmentation strategies were applied to the datasets, such as random cropping, flipping, erasing, and Mixup [72]. During training, the model was supervised using a cross-entropy objective combined with label smoothing. Optimization was performed with the AdamW algorithm. The learning rate was initialized at 0.001 and paired with a weight decay of 0.05. Additionally, a cosine annealing policy was employed to adjust the learning rate dynamically. For a comprehensive and unbiased evaluation, multiple indicators were employed, including parameter count (Params.), Precision (P), Recall (R), and F1-score (F1). Further implementation details can be found in Table 3. To ensure a fair comparison, all baseline models were retrained and evaluated under the same experimental settings described in this study.

### 4.3. Experimental Results and Analysis

All reported classification accuracy metrics are averaged over 10 random runs. The standard deviation was less than 0.3%, indicating statistical stability of the results.

#### 4.3.1. UCM Dataset Experimental Results

**Figure 8:**
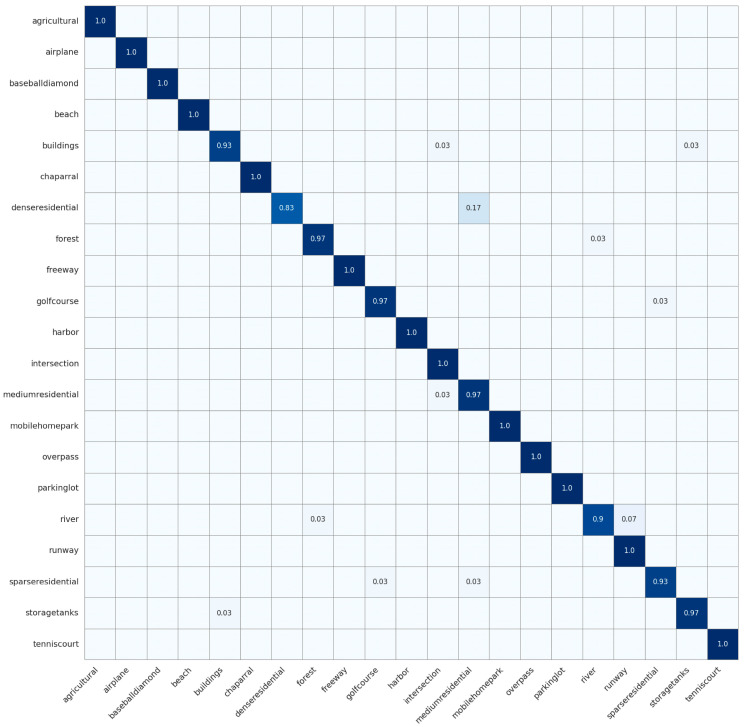
Confusion matrix on the UCM data set.

The experimental results on the UCM dataset are summarized in Table 4. For the experimental results on the UCM, our analysis is as follows:

1. With a training ratio of 70%, our model achieved 97.61% precision, 97.46% recall, and an F1-score of 97.46%. These values were 5.21%, 5.24%, and 5.34% higher than ResNet-101 [73], 5.76%, 5.72%, and 5.84% higher than Swin-B [76], and 2.14%, 2.23%, and 2.21% higher than RSMamba-H [37], respectively. Empirical results revealed that our approach yielded superior performance. By analyzing the experimental results, we found that existing DL methods often overemphasized high-level semantic features of HRRSI while neglecting shallow information that reflected image details and texture features. This challenge prevented a complete and precise capture of the scene’s intricate features, thereby limiting the accuracy of RSSC. Unlike methods that focused exclusively on high-level semantic features, we explicitly incorporated hierarchical feature fusion across shallow (low/mid-level) and deep semantic layers, which allowed the model to capture the scene’s complex features more effectively.
2. Transformer-based models possessed strong global context modeling capabilities but were deficient in local feature extraction, and they had a large number of model parameters. Compared to transformer-based models, such as DeiT-B [74], ViT-L [75], and Swin-B [76], our model achieved improvements of 8.47%, 5.63%, and 5.76% in precision, respectively. Moreover, our model included only 2.6 M parameters, which represented a decrease of 2.9 M, 85.7 M, and 24.9 M compared to the lightweight DeiT-T [74], ViT-B [75], and Swin-T [76] models, respectively. The findings suggested that our model successfully integrated the benefits of both transformer and CNN architectures. We designed the PDSCB to effectively capture features at different scales in the scene using small computational parameters. Based on this, the lightweight SCCA module could more effectively weigh the importance of features and enhance the model’s ability to learn contextual spatial features. In addition, the design of skip connections ensured that feature information at each level could be retained throughout the network’s layer-by-layer depth, thereby preventing the loss of important information in the initial stages and enhancing the model’s capacity to extract features.
3. In the confusion matrix for the UCM dataset shown in Figure 8, LGLMNet achieved 100% accuracy on distinctly textured scenes such as agricultural fields and beaches. However, systematic misclassifications persisted among visually similar categories. Dense residential reached 83% accuracy, with 17% of samples misclassified as medium residential; medium residential achieved 97% accuracy, but 3% of its samples were misidentified as sparse residential; sparse residential reached 93% accuracy, with 3% of its samples misclassified as medium residential and another 3% as golf course. Beyond the residential categories, buildings achieved 93% accuracy, with 3% of samples misassigned to intersection and 3% to storage tanks; river scenes attained 90% accuracy, yet 7% of samples were mistaken for runway. These misclassifications primarily arose from high similarity in geometric layouts and textural patterns, such as comparable rooftop and yard distributions in residential areas, similar grass tones of golf courses and sparse residential yards, and the linear high-contrast structures shared by rivers and runways. Although LGLMNet excelled at distinguishing most scene types, its performance remained limited for categories with highly similar land-cover features and only subtle differences. The standard deviation of the F1 scores across categories is 2.1%, indicating stable model performance over different classes.

#### 4.3.2. AID Dataset Experimental Results

**Figure 9:**
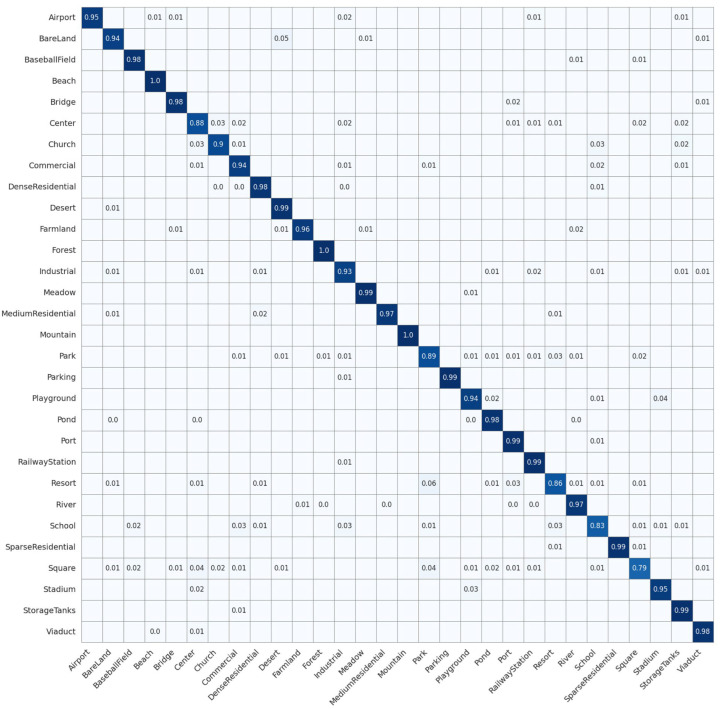
Confusion matrix on the AID dataset.

The experimental results on the AID dataset are summarized in Table 5. For the experimental results on the AID, our analysis is as follows:

1. The AID dataset exhibited greater diversity than the UCM dataset, with images originating from a variety of sensors, thereby rendering scene classification on this dataset more challenging. Under a 50% training split, our method attained 95.29% precision, 95.28% recall, and 95.24% F1 score, which were 4.26%, 4.65%, and 4.43% higher than ResNet-101 [73], 5.45%, 6.27%, and 6.17% higher than Swin-B [76], and 2.32%, 2.77%, and 2.17% higher than RSMamba-H [37], with all three metrics superior to other models. Our model effectively extracted both shallow and high-level features, achieving feature fusion and dimensionality reduction through CLFFB, which significantly enhanced feature distinctiveness and robustness in complex scenes.
2. Compared with models that used a fixed convolutional kernel size and no attention mechanism (e.g., ResNet-18 [71], ResNet-50 [71], ResNet-101 [71]), our model improved precision by 6.59%, 5.85%, and 4.26%, respectively. This indicated that our designed PDSCB could effectively capture the multi-scale features of the scene through the convolution of different receptive fields across multiple branches. At the same time, the SCCA focused on the key spatial locations of the scene and dynamically modified the channel weights using this information. Additionally, the lightweight design of the two modules significantly reduced the parameter count while ensuring the capture of multi-scale contextual features of HRRSI. Compared to models that stacked multiple layers of convolutional kernels, such as ResNet-18, 50, and 101 [71], the number of parameters in our models decreased by 8.6 M, 21.0 M, and 40.0 M, respectively.
3. As shown in the confusion matrix for the AID dataset in Figure 9, LGLMNet maintained at least 95% accuracy across most scene categories and achieved 100% correct classification for uniformly textured, clearly bounded scenes such as beach, forest, and mountain. However, its performance declined for urban scenes with multifunctional subregions. The center category achieved 88% accuracy, with 3% of samples misclassified as church, and 2% each misclassified as commercial, industrial, square, and storage tanks. The square category achieved only 79% accuracy, with misclassifications mainly into park (4%), center (4%), church (2%), baseball field (2%), and pond (2%). The resort category achieved 86% accuracy but was misclassified primarily as park (6%) and port (3%). The school category achieved 83% accuracy, with 3% of samples misclassified as resort, 3% as industrial, and 3% as commercial. These systematic errors stemmed from high similarity in geometric structures and textural patterns among the relevant categories—for example, the center area integrated diverse building functions, the square shared visual features with parking lots and public plazas, resorts shared green-space layouts with parks, and schools shared compositional similarities with commercial and industrial areas. In summary, LGLMNet excelled at distinguishing scenes with simple structures and high separability, but it still faced challenges in urban environments characterized by overlapping fine-grained features and functional diversity. The standard deviation of the per-class F1 scores is 3.5%, slightly higher than that of UCM, reflecting the greater difficulty in classifying certain urban scene categories (e.g., Center, Square) in the AID dataset.

#### 4.3.3. NWPU-45 Dataset Experimental Results

**Figure 10:**
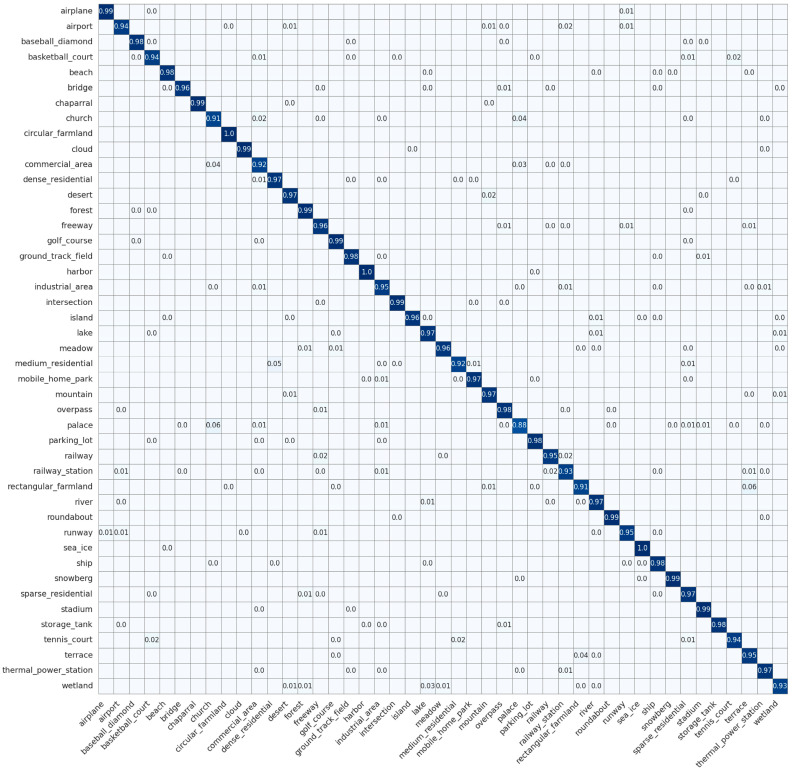
Confusion matrix on the NWPU-45 dataset.

The experimental results on the NWPU-45 dataset are summarized in Table 6. For the experimental results on the NWPU-45, our analysis is as follows:

1. With the most extensive variety of scenes in this experiment in terms of total image classes and scenes, the NWPU-45 dataset also exhibited considerable intragroup differences and significant intergroup similarities, influenced by factors such as spatial resolution, viewpoint, lighting, occlusion, and background variations. This complexity made it more challenging than both the UCM and AID datasets. Our model yielded 96.34% precision, 96.32% recall, and an F1-score of 96.32% under a 70% training split, all of which outperformed other models. Specifically, the three metrics of our model were 3.59%, 3.75%, and 3.76% higher than those of ResNet-101 [73], 2.71%, 4.74%, and 2.76% higher than Swin-B [76], and 1.12%, 1.13%, and 1.14% higher than RSMamb-H [37], respectively. Empirical findings demonstrated that, compared with the DL method that focused solely on high-level semantic features, our method enhanced feature distinctiveness by effectively integrating shallow Lie Group features with high-level semantic features and exhibited a significant advantage in RSSC.
2. RSMamba combined global perception with computational efficiency. The accuracy in the RSSC task was higher than that of the CNN and transformer-based models, and it had a lower parameter count. Our model was 1.47%, 1.31%, and 1.12% more accurate than RSMamba-B [37], RSMamba-L [37], and RSMamba-H [37], respectively, and the number of covariates decreased by 3.8 M, 13.6 M, and 30.5 M, respectively. Empirical evidence confirmed that our model outperformed RSMamba-B [37], RSMamba-L [37], and RSMamba-H [37] in terms of precision. Through PDSCB’s multi-scale feature extraction and the synergy of the SCCA, which enabled the model to learn local and global contextual information, reducing computational cost, the model outperformed RSMamba in accuracy and computational complexity.
3. As shown in Figure 10, experiments on the NWPU-45 dataset demonstrated that LGLMNet achieved at least 95% accuracy in 35 out of 45 scene categories and nearly 100% correct classification in uniformly textured, easily distinguishable classes such as circular farmland, harbor, and sea ice. In stark contrast, classes with complex structures or highly similar features performed relatively poorly: palace and church achieved only 88% and 91% accuracy, respectively, as their repetitive rooftop textures and architectural components were almost indistinguishable; rectangular farmland reached 91% accuracy but was similarly misclassified because its crop-row patterns and field boundaries closely resembled those of the terrace. Overall, LGLMNet effectively extracted discriminative features for the vast majority of scenes but still exhibited blind spots when distinguishing categories with highly overlapping fine-grained land-cover characteristics. The standard deviation of the F1 scores across the 45 categories is 2.8%, demonstrating good overall consistency, though challenges remain for a few structurally similar classes (e.g., Palace/Church).

### 4.4. Computational Complexity and Inference Speed Evaluation

To comprehensively evaluate the deployment efficiency of LGLMNet, we supplement two core metrics: Floating Point Operations (FLOPs) and inference time per image. FLOPs reflect the model’s inherent computational burden, while inference time intuitively demonstrates its practical speed on specific hardware. All tests were conducted on an NVIDIA GeForce RTX 3090 GPU (experimental configuration detailed in Table 2), with a batch size of 1 to simulate the real-world scenario of single-image stream processing.

We compared LGLMNet with several representative baseline models on the AID dataset (image size 600 × 600), and the results are summarized in Table 7. The analysis is as follows:

LGLMNet requires only 1.2 GFLOPs and 3.8 ms of inference time. While already having the lowest parameter count (2.6 M), its computational efficiency remains significantly superior. Compared to the widely used ResNet-50, LGLMNet reduces FLOPs by approximately 70.9% and increases inference speed by about 2.3 times. This primarily benefits from the extensive use of depthwise separable convolutions in the PDSCB module and the efficient design of linear attention in the SCCA module, ensuring low computational complexity at the architectural level.

Compared to the similarly lightweight RSMamba-B model, LGLMNet not only maintains higher classification accuracy (AID accuracy: 95.29% vs. 92.02%) but also offers approximately 26.9% faster inference. Compared to the classic edge-oriented work RSCNet (achieving 2.75 ms inference on CPU), LGLMNet’s performance on GPU is also highly competitive. This fully demonstrates that LGLMNet achieves a better balance across the three dimensions of “accuracy, speed, and parameter count”.

### 4.5. Ablation Experiments

To assess the contribution of each module, ablation experiments were conducted on the UCM dataset using a 70% training split, with consistent training settings to ensure a fair comparison.

#### 4.5.1. The Role of LGML in the Model

The LGML branch focuses on extracting shallow features from HRRSI and building representations using the Lie Group covariance matrix. To evaluate the impact of these features on classification, we conducted ablation studies with and without LGML, comparing the Precision (P) in both cases as summarized in Table 8. Experimental results indicated a 1.10% gain in precision when incorporating LGML, suggesting that it enhanced classification performance and improved the modeling of feature correlations, thereby increasing the interpretability of the model.

#### 4.5.2. The Role of SCCA in the Model

To assess the influence of the SCCA module, we conducted ablation experiments both with and without it, comparing the Precision (P) under both conditions (as shown in Table 9). Incorporating SCCA improved classification precision by 1.06%. These results indicated that SCCA enabled the model to capture spatial–channel correlations more effectively and focus on important feature regions, facilitating RSSC.

#### 4.5.3. The Role of CLFFB in the Model

CLFFB integrates shallow and high-level features within the model. To evaluate its effectiveness, we conducted experiments under two settings: simple channel-wise concatenation and CLFFB-based fusion, with Precision (P) used for comparison (as shown in Table 10). The results showed that incorporating CLFFB increased classification precision by 3.17%, demonstrating its ability to effectively combine shallow Lie Group features with high-level semantic information, thereby enhancing feature discrimination and improving scene recognition performance.

#### 4.5.4. Impact of Different-Sized Depthwise Separable Convolution Kernels on Classification Accuracy and Model Efficiency

To investigate how different sizes of depthwise separable convolution kernels in the PDSCB module affect classification accuracy and model efficiency, we kept the four-branch structure of PDSCB but varied the kernel size combinations of its depthwise convolution branches. The results are shown in Table 11. The combination {5,7,11} (used in our paper) achieves high precision while maintaining relatively low FLOPs. The combination {3,5,7,9,11} (five branches) shows a slight accuracy improvement but a significant increase in FLOPs. Both combinations {3,5,7} and {5,7,9} yield lower accuracy than {5,7,11}. This indicates that in remote sensing scenes, introducing a larger receptive field (11 × 11) is beneficial for capturing large-scale structures (e.g., rivers, roads), and the {5,7,11} combination effectively covers small, medium, and large scales with a controllable computational cost).

#### 4.5.5. Impact of Different Combinations of Shallow Features on Classification Performance

To evaluate the impact of different shallow feature combinations in the LGML branch on classification performance, we tested three configurations: Basic (containing only coordinates, gradients, and color features $Y,C_{b},C_{r}$), +SIFT and LBP (adding SIFT and LBP texture features to Basic), and Full (the complete combination used in our paper, i.e., Equation (2) containing all features). The results are shown in Table 12. It can be seen that the full feature combination (Full) achieves the highest classification precision, improving by 1.8% compared to using only basic features (Basic), and also shows a slight improvement over the +SIFT/LBP configuration. This demonstrates that our comprehensive selection of low/mid-level features is effective and complementary for remote sensing scene representation).

#### 4.5.6. Scale Robustness Test

To verify the model’s robustness to changes in input scale, we conducted an experiment on the UCM test set. Each test image was randomly scaled by a factor s∈[0.5,2.0] before being fed into the model. Table 13 shows the average precision (P) of LGLMNet and ResNet-50 [74] across different scale ranges and their performance drops relative to the original scale (s=1.0). LGLMNet exhibits a smaller average precision drop (2.1%) across scales compared to ResNet-50 (4.7%), suggesting that our model, particularly its multi-scale PDSCB design, offers some robustness to scale variations.

#### 4.5.7. Visualization of Attention

**Figure 11:**
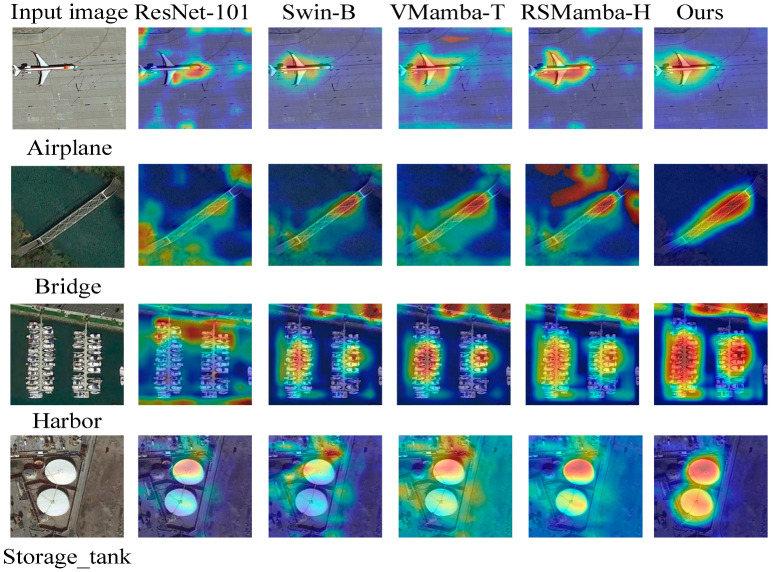
Comparative visualization of Gradient-weighted Class Activation Mapping (Grad-CAM) heatmaps for different models on four scene categories (Airplane, Bridge, Harbor, Storage_tank) from the NWPU-45 dataset. In the heatmaps, red indicates high-attention regions, and blue indicates low-attention regions. Our LGLMNet demonstrates more concentrated and accurate focus regions.

To intuitively understand the decision-making process of the models and validate the effectiveness of the proposed modules, we employed Gradient-weighted Class Activation Mapping (Grad-CAM) to generate visual explanations. A comparative visualization of class activation heatmaps for LGLMNet and several representative baseline models is presented in Figure 11. Four typical scene categories from the NWPU-45 dataset-Airplane, Bridge, Harbor, and Storage_tank-were selected. In the heatmaps, red regions indicate the highest attention of the model when making the classification decision, while blue regions indicate lower attention.

As shown in Figure 11, our LGLMNet accurately focuses on the aircraft fuselage and runway areas in the Airplane scene, covers the bridge structure and its supports in the Bridge scene, and precisely localizes ships/berths and circular tanks in the Harbor and Storage_tank scenes, respectively. In contrast, the attention of ResNet-101 [74] is sometimes less concentrated (e.g., in the Harbor scene). The attention of Swin-B [77] and the state-space model-based VMamba-T [78] and RSMamba-H [37] appears somewhat dispersed or partially shifted in certain scenes (e.g., Storage_tank). These visual results provide intuitive evidence that, by integrating the shallow structural features from LGML, the multi-scale perception of PDSCB, and the collaborative attention of SCCA, LGLMNet can more accurately pinpoint the core discriminative features within a scene. This offers an interpretable basis for its superior classification performance.

## 5. Discussion

### 5.1. The Advantages of Our Method

Through the experiments conducted in Section 4 on the three major public datasets of UCM, AID, and NWPU-45, it is evident that LGLMNet consistently leads in the three core metrics of precision, recall, and F1 score. Compared with methods based on CNN, transformer, and SSM, the model proposed in this paper not only elevates the overall discrimination ability to a new level but also demonstrates stable advantages across all datasets, without experiencing the imbalance of “high precision and low recall” or “high recall and high false positives”. Moreover, its parameter count remains the lowest. The superiority of LGLMNet stems from a triple synergy mechanism: Firstly, the LGML branch can efficiently extract fine-grained features such as shallow texture, color, and contour from HRRSI, and represent these features through symmetric Lie group covariance matrices, deeply exploring the correlations among various features and compensating for the neglect of shallow information by deep networks. Secondly, in the DL branch, the lightweight PDSCB captures multi-scale targets in remote sensing scenes through parallel multi-scale depthwise separable convolutions. At the same time, the SCCA attention mechanism adaptively enhances key information and suppresses redundant features in both spatial and channel dimensions, thereby significantly improving the quality of representation. Finally, the CLFFB module efficiently fuses shallow Lie group features with high-level semantic features, further enhancing the discrimination ability. Thanks to this three-way synergy, LGLMNet not only improves precision but also maintains a high recall rate in complex scenarios, achieving a higher and more balanced F1 score. At the same time, its parameter count is significantly lower than that of existing RSSC methods, providing a feasible solution that combines accuracy and efficiency for edge deployment and large-scale remote sensing applications.

Computational Cost of the LGML Branch: It is acknowledged that the extraction of handcrafted features (SIFT, LBP, Gabor) and covariance computation incurs a fixed preprocessing cost. However, this operation is performed only once per image and is highly parallelizable. The ‘lightweight’ claim primarily refers to the exceptionally low number of trainable parameters (2.6 M) in the deep learning branch and the efficient design of PDSCB/SCCA, which leads to low inference FLOPs and fast runtime, as evidenced in Table 6. The fixed cost of LGML feature extraction is a trade-off for gaining robust shallow representations.

### 5.2. The Limitations of Our Method

Although the experimental results of the proposed LGLMNet demonstrate superior performance across the three datasets, certain limitations remain that warrant further investigation and improvement in future research.

#### 5.2.1. The Reliance on Prior Knowledge

In the LGML branch, the selection of shallow features and the construction of their Lie group covariance matrices largely rely on professional knowledge in the field of remote sensing and digital image processing. The shallow Lie Group feature vectors adopted in this study were formed based on previous work and extensive experimental verification. Therefore, non-professional readers may still face certain barriers if they want to understand and reproduce this method deeply. Additionally, different application scenarios usually require targeted adjustments to the feature extraction strategy, and this process is also highly dependent on the user’s domain experience, which, to some extent, limits the wide promotion of the method proposed in this paper.

#### 5.2.2. Generalization and Real-Time Challenges in Practical Applications

Although LGLMNet performs well on public datasets, real-world remote sensing image scenes are far more complex than these “clean” data: due to the inherent differences in spectral response, radiometric correction, and spatial resolution among various satellites or aerial sensors, the same scene will show significant inter-sensor distribution differences in different data sources; if the model has not undergone cross-sensor or multi-source generalization training, such distribution differences will significantly reduce its recognition accuracy on new data; seasonal changes, vegetation phenology, and atmospheric conditions will also cause the same ground object to present completely different spectral-textural features in the time series, thereby affecting classification accuracy. At the same time, actual deployment often requires real-time or near real-time inference, and the computing power and memory of edge devices such as GPUs, FPGAs, or MCUs are limited. Even if the model is relatively lightweight, it still needs to rely on quantization, pruning, or distillation and other means to meet the constraints of second-level response and low energy consumption.

### 5.3. Future Work

In summary, our subsequent research will focus on the following tasks: In terms of generalization, we plan to introduce a remote sensing dataset that covers multiple remote sensing platforms and has more diverse scenes for model training. To achieve real-time processing, we need to optimize in terms of computational efficiency. We plan to optimize the SCCA attention mechanism’s implementation, explore lightweight backbone networks, and investigate more efficient feature fusion methods. Additionally, we aim to design more efficient network structures to improve classification accuracy while minimizing the number of parameters and computational complexity.

## 6. Conclusions

In this work, we proposed LGLMNet, a lightweight multi-scale convolutional network that integrates LGML and DL. The model features two parallel branches of LGML and DL to extract shallow Lie Group features and high-level semantic features, respectively. To enhance multi-scale perceptual capabilities, we designed a lightweight PDSCB. Additionally, we introduced an SCCA, which unifies spatial and channel contexts to improve global modeling capabilities. For feature fusion, the CLFFB combines shallow and high-level information to enhance feature expression abilities and achieve dimensionality reduction. Experiments conducted on three challenging remote sensing datasets validated the model’s superior performance presented in this paper. Nevertheless, there is still potential for further improvement in the fusion of LGML and DL.

In the future, our research will continue to explore deep fusion pathways of LGML, integrating attention mechanisms, feature extraction, and other approaches to optimize model performance. Moreover, we will explicitly explore “class imbalance in remote sensing scene classification, multiple platforms, and more diverse scenarios” as a dedicated topic.

## Figures and Tables

**Table 1:** Hyperparameter Settings for Each Module in Figure 5.

Module	Hyperparameter Symbol	Meaning	Specific Value
Figure 5a Deep Learning Branch	C_stem_	Output channels of Stem module	32
	C_i_	Output channels of Stage i (i = 1, 2, 3, 4)	C_1_ = 64, C_2_ = 128, C_3_ = 256, C_4_ = 512
	N_i_	Number of Basic Blocks in Stage i	N_1_ = N_2_ = N_3_ = N_4_ = 2
		Downsampling method	Max pooling, kernel size 2 × 2, stride 2
Figure 5b Stem Module	C_in_	Input channels	3 (e.g., RGB image)
	K	Depthwise convolution kernel size	3 × 3
	S	Convolution stride	1
	P	Padding	1
		Output channels	C_stem_ = 32
Figure 5c Basic Block	C	Input/output channels	Matches the internal channel number of the corresponding Stage (i.e., C = 64 in Stage 1, C = 128 in Stage 2, C = 256 in Stage 3, C = 512 in Stage 4)
Figure 5d PDSCB Module	K_pre_	Kernel size of the preceding 3 × 3 depthwise convolution	3
		Number of groups for channel split	4 (each group has C/4 channels)
	K_2_, K_3_, K_4_	Depthwise convolution kernel sizes in parallel branches	K_2_ = 5, K_3_ = 7, K_4_ = 11
		Padding for each branch	2, 3, 5 respectively (to keep spatial size unchanged)
		Output channels of the 1 × 1 convolution after concatenation	C
		Residual connection	Input F added to the PDSCB output

**Table 2:** Key characteristics of UCM, AID, and NWPU-45 datasets.

Dataset	Release Year	Number of Classes	Number of Images	Image Size	Resolution (m)	Train Split
UCM	2010	21	2100	256 × 256	0.3	70%
AID	2017	30	10,000	600 × 600	0.5–8	50%
NWPU-45	2017	45	31,500	256 × 256	0.2–30	70%

**Table 3:** Experimental settings and environment configuration.

Item	Content
Hardware Environment	
CPU	Xeon Platinum 8362 with 2.8 GHz × 32
GPU	NVIDIA GeForce RTX 3090
Operating system	CentOS 7.8 64-bit
Memory	24 GB
Software Environment	
Operating System	CentOS 7.8 64-bit
Python	3.8
CUDA	11.8
Deep Learning Framework	PyTorch 2.0.0
Training Hyperparameters	
Optimizer	AdamW (β_1_ = 0.9, β_2_ = 0.999, eps = 1 × 10^−8^)
Initial Learning rate	0.001
Weight decay	0.05
Learning Rate Schedule	Cosine Annealing, with a minimum learning rate of 1 × 10^−5^
Training Epochs	200
Batch	32
Data Augmentation	Random cropping, random horizontal flipping, random erasing, Mixup (α = 0.2)
Loss Function	Label Smoothing Cross-Entropy Loss (label smoothing factor = 0.1)

**Table 4:** Performance comparison on UCM with 70% training data.

Method	Params. (M)	UCM (70%)		
		P (%)	R (%)	F1 (%)
ResNet-18 [73]	11.2	87.98	87.46	87.40
ResNet-50 [73]	23.6	91.99	91.74	91.65
ResNet-101 [73]	42.6	92.40	92.22	92.12
DeiT-T [74]	5.5	86.92	86.66	86.53
DeiT-S [74]	21.7	88.95	88.41	88.41
DeiT-B [74]	85.5	89.14	88.73	88.70
ViT-B [75]	88.3	91.09	90.79	90.77
ViT-L [75]	303.0	91.98	91.32	91.26
Swin-T [76]	27.5	90.87	90.63	90.40
Swin-S [76]	48.9	91.08	90.95	90.82
Swin-B [76]	86.8	91.85	91.74	91.62
Vim-Ti [77]	7.0	89.06	88.73	88.68
VMamba-T [78]	30.0	93.14	92.85	92.81
RSMamba-B [37]	6.4	94.14	93.97	93.88
RSMamba-L [37]	16.2	95.03	94.76	94.74
RSMamba-H [37]	33.1	95.47	95.23	95.25
LGLMNet (Ours)	2.6	97.61 ± 0.15	97.46 ± 0.18	97.46 ± 0.15

**Table 5:** Performance comparison on AID with 50% training data.

Method	Params. (M)	AID (50%)		
		P (%)	R (%)	F1 (%)
ResNet-18 [73]	11.2	88.70	88.17	88.30
ResNet-50 [73]	23.6	89.44	88.66	88.87
ResNet-101 [73]	42.6	91.03	90.63	90.81
DeiT-T [74]	5.5	85.23	84.52	84.52
DeiT-S [74]	21.7	85.88	85.19	85.34
DeiT-B [74]	85.5	87.32	86.07	86.07
ViT-B [75]	88.3	89.39	88.65	88.86
ViT-L [75]	303.0	90.19	88.86	89.17
Swin-T [76]	27.5	86.49	85.66	85.77
Swin-S [76]	48.9	87.50	86.80	86.89
Swin-B [76]	86.8	89.84	89.01	89.07
Vim-Ti [77]	7.0	87.76	86.98	87.13
VMamba-T [78]	30.0	91.59	90.94	91.10
RSMamba-B [37]	6.4	92.02	91.53	91.66
RSMamba-L [37]	16.2	92.31	91.75	91.90
RSMamba-H [37]	33.1	92.97	92.51	92.63
LGLMNet (Ours)	2.6	95.29 ± 0.18	95.28 ± 0.19	95.24 ± 0.18

**Table 6:** Performance comparison on NWPU-45 with 70% training data.

Method	Params. (M)	NWPU-45 (70%)		
		P (%)	R (%)	F1 (%)
ResNet-18 [73]	11.2	88.73	88.44	88.45
ResNet-50 [73]	23.6	92.67	92.47	92.47
ResNet-101 [73]	42.6	92.75	92.57	92.56
DeiT-T [74]	5.5	87.66	86.78	86.79
DeiT-S [74]	21.7	88.21	87.47	87.43
DeiT-B [74]	85.5	89.04	88.62	88.65
ViT-B [75]	88.3	88.84	88.65	88.62
ViT-L [75]	303.0	91.22	91.08	91.04
Swin-T [76]	27.5	90.15	90.06	90.06
Swin-S [76]	48.9	92.05	91.88	91.84
Swin-B [76]	86.8	93.63	91.58	93.56
Vim-Ti [77]	7.0	89.24	89.02	88.97
VMamba-T [78]	30.0	93.97	93.96	93.94
RSMamba-B [37]	6.4	94.87	94.87	94.84
RSMamba-L [37]	16.2	95.03	95.05	95.02
RSMamba-H [37]	33.1	95.22	95.19	95.18
LGLMNet (Ours)	2.6	96.34 ± 0.12	96.32 ± 0.14	96.32 ± 0.12

**Table 7:** Comparison of model efficiency on the AID dataset.

Models	Params (M)	FLOPs (G)	Inference Time (ms)	Speedup vs. ResNet-50
ResNet-50 [73]	23.6	4.12	8.7	1.00×
Swin-T [76]	27.5	5.83	12.3	0.71×
RSMamba-B [37]	6.4	1.95	5.2	1.67×
Ours	2.6	1.08	3.8	2.29×

**Table 8:** Assessment of the impact of LGML. (**↑** The upward arrow indicates an increase.).

Models	P (%)
Without the LGML	96.51
Ours	97.61
Change	1.10 **↑**

**Table 9:** Assessing the Impact of SCCA Attention Mechanisms (**↑** The upward arrow indicates an increase).

Models	P (%)
Without the SCCA	96.55
Ours	97.61
Change	1.06 **↑**

**Table 10:** Assessing the Impact of CLFFB (**↑** The upward arrow indicates an increase).

Models	P (%)
Use the concat	94.44
Use the CLFFB	97.61
Change	3.17 **↑**

**Table 11:** Impact of Depthwise Convolution Kernel Sizes in PDSCB.

Kerne Size Combination	P (%)	FLOPs (G)
{3,5,7}	96.88	1.02
{5,7,9}	97.12	1.05
{5,7,11}	97.61	1.08
{3,5,7,9,11}	97.73	1.25

**Table 12:** Impact of Shallow Feature Combinations.

Feature Configuration	P (%)
Basic	95.62
+SIFT/LBP	96.87
Full	97.61

**Table 13:** Performance under scale variations on UCM.

Scale Range	LGLMNet P (%)	Drop (%)	ResNet-50 P (%)	Drop (%)
s=1.0 (Original)	97.61	-	91.99	-
s∈[0.5,0.8)	96.12	1.49	88.45	3.54
s∈[0.8,1.2]	97.35	0.26	91.67	0.32
s∈(1.2,2.0]	94.88	2.73	86.01	5.68
Average Drop	-	2.1	-	4.7

## Data Availability

The data presented in this study are openly available in UC Merced Land Use Dataset at https://www.kaggle.com/datasets/abdulhasibuddin/uc-merced-land-use-dataset (accessed on 9 June 2025), NWPU-RESISC45 Dataset at https://gcheng-nwpu.github.io/#Datasets (accessed on 9 June 2025), and the Aerial Image Dataset (AID) at https://captain-whu.github.io/AID/ (accessed on 9 June 2025).

## Abbreviations

AID	Aerial Image Dataset
AdamW	Adaptive Moment Estimation with Decoupled Weight Decay
BoVW	Bag-of-Visual-Words
BN	Batch Normalization
CNN	Convolutional Neural Network
CLFFB	Cross-Layer Feature Fusion Block
DL	Deep Learning
DeiT	Data-efficient image Transformers
F1	F1 score
FV	Fisher Vector
GAP	Global Average Pooling
GN	Group Normalization
HRRSI	High-resolution Remote Sensing Images
LBP	Local Binary Pattern
LGML	Lie Group Machine Learning
LGLMNet	Lie Group lightweight multi-scale network
MLP	Multilayer Perceptron
PTM	Probabilistic Topic Models
PDSCB	Parallel Depthwise Separable Convolution Block
PWConv	Pointwise Convolutions
RSSC	Remote Sensing Scene Classification
ReLU	Rectified Linear Unit
SIFT	Scale-Invariant Feature Transform
SiLU	Sigmoid Linear Unit
SCCA	Spatial-Channel Collaborative Attention Mechanism
Swin	Swin Transformer
SSM	State Space Model
ViT	Vision Transformer

## References

[1] Xu C., Shu J., Wang J., Wang Z. (2024). Remote sensing scene classification based on contextual attention mechanism of lie group space. Int. J. Remote Sens..

[2] Cheng G., Xie X., Han J., Guo L., Xia G.-S. (2020). Remote sensing image scene classification meets deep learning: Challenges, methods, benchmarks, and opportunities. IEEE J. Sel. Top. Appl. Earth Obs. Remote Sens..

[3] Li D., Wang M., Dong Z., Shen X., Shi L. (2017). Earth observation brain (EOB): An intelligent earth observation system. Geo-Spat. Inf. Sci..

[4] Liu C., Chen K., Zhang H., Qi Z., Zou Z., Shi Z. (2024). Change-agent: Towards interactive comprehensive remote sensing change interpretation and analysis. IEEE Trans. Geosci. Remote Sens..

[5] Ye Z., Zhang Y., Zhang J., Li W., Bai L. (2024). A multiscale incremental learning network for remote sensing scene classification. IEEE Trans. Geosci. Remote Sens..

[6] Xu Z., Zhou Y., Wang S., Wang L., Li F., Wang S., Wang Z. (2020). A novel intelligent classification method for urban green space based on high-resolution remote sensing images. Remote Sens..

[7] Yu D., Fang C. (2023). Urban remote sensing with spatial big data: A review and renewed perspective of urban studies in recent decades. Remote Sens..

[8] Cheng G., Guo L., Zhao T., Han J., Li H., Fang J. (2013). Automatic landslide detection from remote-sensing imagery using a scene classification method based on BoVW and pLSA. Int. J. Remote Sens..

[9] Zhong C., Liu Y., Gao P., Chen W., Li H., Hou Y., Nuremanguli T., Ma H. (2020). Landslide mapping with remote sensing: Challenges and opportunities. Int. J. Remote Sens..

[10] Cheng G., Zhou P., Han J. (2016). Learning rotation-invariant convolutional neural networks for object detection in VHR optical remote sensing images. IEEE Trans. Geosci. Remote Sens..

[11] Lin Z., He Z., Wang X., Liang H., Su W., Tan J., Xie S. (2024). Cross-scale hybrid Gaussian attention network for object detection in remote sensing images. IEEE Geosci. Remote Sens. Lett..

[12] Huang X., Wen D., Li J., Qin R. (2017). Multi-level monitoring of subtle urban changes for the megacities of China using high-resolution multi-view satellite imagery. Remote Sens. Environ..

[13] Zhu J., Li L. (2025). Advancements in image classification for environmental monitoring using AI. Front. Environ. Sci..

[14] Javed A., Kim T., Lee C., Oh J., Han Y. (2023). Deep learning-based detection of urban forest cover change along with overall urban changes using very-high-resolution satellite images. Remote Sens..

[15] Li Z., Chen B., Wu S., Su M., Chen J.M., Xu B. (2024). Deep learning for urban land use category classification: A review and experimental assessment. Remote Sens. Environ..

[16] Ma W., Guo Y., Zhu H., Yi X., Zhao W., Wu Y., Hou B., Jiao L. (2024). Intra-and intersource interactive representation learning network for remote sensing images classification. IEEE Trans. Geosci. Remote Sens..

[17] Paheding S., Saleem A., Siddiqui M.F.H., Rawashdeh N., Essa A., Reyes A.A. (2024). Advancing horizons in remote sensing: A comprehensive survey of deep learning models and applications in image classification and beyond. Neural Comput. Appl..

[18] Xu Y., Bi H., Yu H., Lu W., Li P., Li X., Sun X. (2024). Attention-based contrastive learning for few-shot remote sensing image classification. IEEE Trans. Geosci. Remote Sens..

[19] Zhang W., Tang P., Zhao L. (2019). Remote sensing image scene classification using CNN-CapsNet. Remote Sens..

[20] Zhang W., Jiao L., Liu F., Liu J., Cui Z. (2022). LHNet: Laplacian convolutional block for remote sensing image scene classification. IEEE Trans. Geosci. Remote Sens..

[21] Xu C., Zhu G., Shu J. (2021). A lightweight and robust lie group-convolutional neural networks joint representation for remote sensing scene classification. IEEE Trans. Geosci. Remote Sens..

[22] Cao R., Fang L., Lu T., He N. (2020). Self-attention-based deep feature fusion for remote sensing scene classification. IEEE Geosci. Remote Sens. Lett..

[23] Vaswani A., Shazeer N., Parmar N., Uszkoreit J., Jones L., Gomez A.N., Kaiser Ł., Polosukhin I. (2017). Attention is all you need. arXiv.

[24] Bazi Y., Bashmal L., Rahhal M.M.A., Dayil R.A., Ajlan N.A. (2021). Vision transformers for remote sensing image classification. Remote Sens..

[25] Lv P., Wu W., Zhong Y., Du F., Zhang L. (2022). SCViT: A spatial-channel feature preserving vision transformer for remote sensing image scene classification. IEEE Trans. Geosci. Remote Sens..

[26] Sha Z., Li J. (2022). MITformer: A multiinstance vision transformer for remote sensing scene classification. IEEE Geosci. Remote Sens. Lett..

[27] Chen S.-B., Wei Q.-S., Wang W.-Z., Tang J., Luo B., Wang Z.-Y. (2021). Remote sensing scene classification via multi-branch local attention network. IEEE Trans. Image Process..

[28] Tong L., Liu J., Du B. (2025). SceneFormer: Neural architecture search of Transformers for remote sensing scene classification. IEEE Trans. Geosci. Remote Sens..

[29] Lowe D.G. (2004). Distinctive image features from scale-invariant keypoints. Int. J. Comput. Vis..

[30] Barburiceanu S., Terebes R., Meza S. (2021). 3D texture feature extraction and classification using GLCM and LBP-based descriptors. Appl. Sci..

[31] Bi Q., Qin K., Zhang H., Xie J., Li Z., Xu K. (2019). APDC-Net: Attention pooling-based convolutional network for aerial scene classification. IEEE Geosci. Remote Sens. Lett..

[32] Martins V.S., Kaleita A.L., Gelder B.K., da Silveira H.L., Abe C.A. (2020). Exploring multiscale object-based convolutional neural network (multi-OCNN) for remote sensing image classification at high spatial resolution. ISPRS J. Photogramm. Remote Sens..

[33] Chen L., Li S., Bai Q., Yang J., Jiang S., Miao Y. (2021). Review of image classification algorithms based on convolutional neural networks. Remote Sens..

[34] Khan S., Naseer M., Hayat M., Zamir S.W., Khan F.S., Shah M. (2022). Transformers in vision: A survey. ACM Comput. Surv. (CSUR).

[35] Chen Z., Yang J., Feng Z., Chen L., Li L. (2023). BiShuffleNeXt: A lightweight bi-path network for remote sensing scene classification. Measurement.

[36] Xu C., Shu J., Wang Z., Wang J. (2024). A Scene Classification Model Based on Global-Local Features and Attention in Lie Group Space. Remote Sens..

[37] Chen K., Chen B., Liu C., Li W., Zou Z., Shi Z. (2024). Rsmamba: Remote sensing image classification with state space model. IEEE Geosci. Remote Sens. Lett..

[38] Xu C., Zhu G., Shu J. (2022). A combination of lie group machine learning and deep learning for remote sensing scene classification using multi-layer heterogeneous feature extraction and fusion. Remote Sens..

[39] Thapa A., Horanont T., Neupane B., Aryal J. (2023). Deep learning for remote sensing image scene classification: A review and meta-analysis. Remote Sens..

[40] Ojala T., Pietikäinen M., Harwood D. (1996). A comparative study of texture measures with classification based on featured distributions. Pattern Recognit..

[41] Huang L., Chen C., Li W., Du Q. (2016). Remote sensing image scene classification using multi-scale completed local binary patterns and fisher vectors. Remote Sens..

[42] Risojević V., Momić S., Babić Z. (2011). Gabor descriptors for aerial image classification. Proceedings of the Adaptive and Natural Computing Algorithms: 10th International Conference, ICANNGA 2011, Ljubljana, Slovenia, 14–16 April 2011.

[43] Kabir S., He D., Sanusi M., Wan Hussina W. (2010). Texture analysis of IKONOS satellite imagery for urban land use and land cover classification. Imaging Sci. J..

[44] Lu D., Weng Q. (2007). A survey of image classification methods and techniques for improving classification performance. Int. J. Remote Sens..

[45] Amiri K., Farah M., Leloglu U.M. (2020). BoVSG: Bag of visual SubGraphs for remote sensing scene classification. Int. J. Remote Sens..

[46] Zhao B., Zhong Y., Xia G.-S., Zhang L. (2015). Dirichlet-derived multiple topic scene classification model for high spatial resolution remote sensing imagery. IEEE Trans. Geosci. Remote Sens..

[47] Zhao B., Zhong Y., Zhang L., Huang B. (2016). The Fisher kernel coding framework for high spatial resolution scene classification. Remote Sens..

[48] Zhu Q., Zhong Y., Zhao B., Xia G.-S., Zhang L. (2016). Bag-of-visual-words scene classifier with local and global features for high spatial resolution remote sensing imagery. IEEE Geosci. Remote Sens. Lett..

[49] Zhong Y., Zhu Q., Zhang L. (2015). Scene classification based on the multifeature fusion probabilistic topic model for high spatial resolution remote sensing imagery. IEEE Trans. Geosci. Remote Sens..

[50] Zhong Y., Fei F., Liu Y., Zhao B., Jiao H., Zhang L. (2017). SatCNN: Satellite image dataset classification using agile convolutional neural networks. Remote Sens. Lett..

[51] Peng C., Li Y., Jiao L., Shang R. (2020). Efficient convolutional neural architecture search for remote sensing image scene classification. IEEE Trans. Geosci. Remote Sens..

[52] Song P., Li J., An Z., Fan H., Fan L. (2022). CTMFNet: CNN and transformer multiscale fusion network of remote sensing urban scene imagery. IEEE Trans. Geosci. Remote Sens..

[53] Zhao Y., Liang J., Huang S., Huang P. (2024). Hierarchical deep features progressive aggregation for remote sensing images scene classification. IEEE J. Sel. Top. Appl. Earth Obs. Remote Sens..

[54] Yu H., Xu Z., Zheng K., Hong D., Yang H., Song M. (2022). MSTNet: A multilevel spectral–spatial transformer network for hyperspectral image classification. IEEE Trans. Geosci. Remote Sens..

[55] Chen Y., Li Y., Mao H., Chai X., Jiao L. (2023). A novel deep nearest neighbor neural network for few-shot remote sensing image scene classification. Remote Sens..

[56] Zhao Z., Li J., Luo Z., Li J., Chen C. (2020). Remote sensing image scene classification based on an enhanced attention module. IEEE Geosci. Remote Sens. Lett..

[57] Szegedy C., Liu W., Jia Y., Sermanet P., Reed S., Anguelov D., Erhan D., Vanhoucke V., Rabinovich A. Going deeper with convolutions. Proceedings of the IEEE Conference on Computer Vision and Pattern Recognition.

[58] Xu C., Zhu G., Shu J. (2020). Robust joint representation of intrinsic mean and kernel function of lie group for remote sensing scene classification. IEEE Geosci. Remote Sens. Lett..

[59] Xu C., Zhu G., Shu J. (2020). A lightweight intrinsic mean for remote sensing classification with lie group kernel function. IEEE Geosci. Remote Sens. Lett..

[60] Lin D., Grimson E., Fisher J. Learning visual flows: A Lie algebraic approach. Proceedings of the 2009 IEEE Conference on Computer Vision and Pattern Recognition.

[61] Huang Z., Wan C., Probst T., Van Gool L. Deep learning on lie groups for skeleton-based action recognition. Proceedings of the IEEE Conference on Computer Vision and Pattern Recognition.

[62] Xu C., Govindarajan L.N., Zhang Y., Cheng L. (2017). Lie-x: Depth image based articulated object pose estimation, tracking, and action recognition on lie groups. Int. J. Comput. Vision.

[63] Al-Kadi O.S. (2017). A gabor filter texture analysis approach for histopathological brain tumor subtype discrimination. arXiv.

[64] Recio J.A.R., Fernandez L.A.R., Fernández-Sarriá A. (2005). Use of Gabor filters for texture classification of digital images. Física Tierra.

[65] Ganesan P., Rajini V., Sathish B., Kalist V., Basha S.K. (2015). Satellite image segmentation based on YCbCr color space. Indian J. Sci. Technol..

[66] Cai X., Lai Q., Wang Y., Wang W., Sun Z., Yao Y. (2024). Poly Kernel Inception Network for Remote Sensing Detection. arXiv.

[67] Si Y., Xu H., Zhu X., Zhang W., Dong Y., Chen Y., Li H. (2025). SCSA: Exploring the synergistic effects between spatial and channel attention. Neurocomputing.

[68] Yang Y., Newsam S. Bag-of-visual-words and spatial extensions for land-use classification. Proceedings of the 18th SIGSPATIAL International Conference on Advances in Geographic Information Systems.

[69] Choromanski K., Likhosherstov V., Dohan D., Song X., Gane A., Sarlos T., Hawkins P., Davis J., Mohiuddin A., Kaiser L. (2020). Rethinking attention with performers. arXiv.

[70] Xia G.-S., Hu J., Hu F., Shi B., Bai X., Zhong Y., Zhang L., Lu X. (2017). AID: A benchmark data set for performance evaluation of aerial scene classification. IEEE Trans. Geosci. Remote Sens..

[71] Cheng G., Han J., Lu X. (2017). Remote sensing image scene classification: Benchmark and state of the art. Proc. IEEE.

[72] Zhang H., Cisse M., Dauphin Y.N., Lopez-Paz D. (2017). mixup: Beyond empirical risk minimization. arXiv.

[73] He K., Zhang X., Ren S., Sun J. Deep residual learning for image recognition. Proceedings of the IEEE Conference on Computer Vision and Pattern Recognition.

[74] Touvron H., Cord M., Douze M., Massa F., Sablayrolles A., Jégou H. Training data-efficient image transformers & distillation through attention. Proceedings of the International Conference on Machine Learning.

[75] Dosovitskiy A., Beyer L., Kolesnikov A., Weissenborn D., Zhai X., Unterthiner T., Dehghani M., Minderer M., Heigold G., Gelly S. (2020). An image is worth 16x16 words: Transformers for image recognition at scale. arXiv.

[76] Liu Z., Lin Y., Cao Y., Hu H., Wei Y., Zhang Z., Lin S., Guo B. Swin transformer: Hierarchical vision transformer using shifted windows. Proceedings of the IEEE/CVF International Conference on Computer Vision.

[77] Zhu L., Liao B., Zhang Q., Wang X., Liu W., Wang X. (2024). Vision Mamba: Efficient Visual Representation Learning with Bidirectional State Space Model. arXiv.

[78] Liu Y., Tian Y., Zhao Y., Yu H., Xie L., Wang Y., Ye Q., Liu Y. (2024). VMamba: Visual State Space Model. arXiv.

